# Evaluating the impact of oral hygiene instruction and digital oral health education within cardiac rehabilitation clinics: A protocol for a novel, dual centre, parallel randomised controlled trial

**DOI:** 10.1371/journal.pone.0306882

**Published:** 2024-07-11

**Authors:** Lauren Church, Axel Spahr, Simone Marschner, Janet Wallace, Clara Chow, Shalinie King

**Affiliations:** 1 The University of Sydney Dental School, The University of Sydney, Sydney, New South Wales, Australia; 2 Westmead Applied Research Centre, The University of Sydney, Westmead, New South Wales, Australia; 3 School of Health Sciences, Oral Health, The University of Newcastle, Ourimbah, New South Wales, Australia; 4 The University of Sydney Medical School, The University of Sydney, Sydney, New South Wales, Australia; Mersin University: Mersin Universitesi, TÜRKIYE

## Abstract

**Introduction:**

Diseases of the periodontal tissues including gingivitis and periodontitis can affect up to 90% and 50% of the population respectively. These conditions are multifactorial inflammatory conditions involving a dysbiotic biofilm that, if left untreated, can lead to the destruction of the supporting structures of the teeth and have significant systemic implications, specifically on cardiovascular health. The elevation of inflammatory markers, particularly high-sensitive C-reactive protein (hsCRP), are strongly associated with an increased risk of atherosclerosis, a key risk factor for cardiovascular disease (CVD). HsCRP as well as other inflammatory markers can be detected in blood samples as early as 21 days after ceasing toothbrushing, due to the immune response to stagnant oral biofilm. The most effective way to ensure oral biofilm cannot remain on oral tissues, thus preventing periodontitis and reducing inflammatory CVD risk, is with good oral hygiene. The primary aim of this study is to assess whether individualised oral hygiene instruction (OHI) partnered with a digital oral health education (DOHE) package can improve the oral health of patients living with CVD.

**Methods and analysis:**

A total of 165 participants will be recruited from the Westmead and Blacktown Mt Druitt cardiac rehabilitation out-patient clinics into this dual centre, single blind, parallel design, randomised controlled trial. A baseline oral health clinical examination will be completed, followed by a self-report questionnaire before they are randomised in a 1:1:1 ratio into one of 3 arms as follows: individualised OHI partnered with DOHE (Group A), (Group B) DOHE only (Group B), and control/usual care (no oral health education) (Group C). Groups will have their intervention repeated at the 6-week follow-up. After completing the 12-week follow-up, Group B and Group C will receive tailored OHI. Group C will also receive the DOHE package. The primary outcome is the change in approximal plaque index score between baseline and 6-week follow up.

**Ethics and dissemination:**

The study has been approved by the Western Sydney Local Health District Human Ethics Committee 2023/ETH00516. Results will be published in peer-reviewed journals and presented at conferences.

**Trial registration number:**

ACTRN12623000449639p ANZCTR: https://www.anzctr.org.au/.

## Introduction

### Periodontal disease

All dentated individuals are susceptible to periodontal disease [1] including gingivitis, affecting up to 90% of any population; and periodontitis affecting close to 50% of the global population [2]. Periodontitis is defined as a multifactorial inflammatory process involving dysbiotic biofilm [3], a well organised colony of bacteria [1]; that, if left untreated, may lead to destruction of the supporting structures of the teeth including connective tissue, periodontal ligament fibres, and alveolar bone [4, 5].

Gingivitis, a reversible inflammatory condition of gingival tissues, initiates within 24-48hrs after ceasing toothbrushing [6]. Once the biofilm makes contact with gingival tissues the innate immune response is activated [7].

Within 3 weeks post cessation of toothbrushing, the predominately supragingival, aerobic gram-positive biofilm will slowly migrate deeper into the gingival sulcus and transform into mostly anaerobic gram-negative [1, 8] and an increase in inflammatory markers high-sensitive C-reactive protein (hsCRP) and interluken-6 (IL-6) will be detected in blood samples [9].

At this stage in the disease process, the immune system is at its most powerful. Responding to the bacteria and their exo- and endotoxins, it releases proinflammatory cytokines interleukin-1 alpha (IL-1α), interleukin-1 beta (IL-1β), tumour necrosis factor-alpha (TNF-α), interleukin-8 (IL-8), interleukin-1 receptor antagonist (IL-1ra), interleukin-10 (IL-10), and interleukin-12 (IL-12), matrix metalloproteinases (MMPs) and prostaglandin E2 (PGE_2_) as well as T and B cell lymphocytes, polymorphonuclear leukocytes (PMNs). For most people, the battle of bacteria and immune response remains balanced, and if tooth brushing is reintroduced, the disease state resolves without any permanent damage to oral tissues [1, 8]. However, the immune response intensity in susceptible individuals can lead periodontitis, initiating irreversible destruction of connective tissue, periodontal ligament fibres, and alveolar bone [6].

Currently, periodontitis affects 30% of adult Australians [10] and is the sixth most prevalent chronic condition worldwide [11] increasing in incidence by 8.44% over the last 20 years [12]. The irreversible damage periodontitis causes can have severe implications on quality of life (QoL) as it can involve tooth mobility and eventual tooth loss; affecting speech, eating, and causing psychological damage [13, 14].

### Periodontal inflammation and cardiovascular disease

The elevation of hsCRP and IL-6 is strongly associated with an increased risk of atherosclerosis [15], a leading risk factor for cardiovascular disease (CVD) [16]. Atherosclerosis is developed from matured atherosclerotic plaque forming on blood vessel walls [17]. This fibro-lipid structure consists of key immune inflammatory cells such as leukocytes and markers including cytokines, chemoattractants, and MMPs [18]. Whilst inflammation plays a key role in all stages of atherosclerosis [19], oral biofilm has been shown to influence atherosclerotic plaque and has been hypothesized to trigger its formation [18, 20] highlighting its involvement in CVD [21–23].

In 2021, CVD was the leading cause of death in Australia [24] and is responsible for 32% of deaths and 38% of premature deaths globally [25]. Patients living with CVD are at high risk of having a cardiovascular event when inflammatory markers are elevated [26, 27], especially after surviving a myocardial infarction (MI) [28]. Untreated periodontitis contributes to this inflammatory risk [29]. Therefore, reducing this inflammatory comorbidity may help prevent poor CVD outcomes.

### Prevention of periodontal disease

The most important and effective way to prevent periodontal disease is with an optimal oral hygiene routine [30], followed by regular dental check-ups and hygiene appointments, as well as the absence or control of modifying risk factors such as smoking [31] and diabetes [32]. Poor oral hygiene habits, defined as never or rarely brushing teeth, have been shown to increase the risk of a cardiovascular event significantly (HR 1.7, 95% CI 1.3 to 2.3; P<0.001) [33]. Incorporating just one toothbrushing session per day is associated with a 9% risk reduction of a cardiovascular event [34]. The recommended oral hygiene routine includes brushing twice daily, preferably with an electric oscillating/rotating power toothbrush [35–37], for at least two minutes with fluoridated toothpaste [38, 39]. Additionally, as toothbrushes cannot reach all tooth surfaces, particularly in between the teeth, interdental cleaning products such as interdental brushes should be used before brushing at least once daily [40–42].

### Oral hygiene promotion studies

Essential to good oral hygiene and the prevention of oral and systemic disease, is education [43]. Currently, the most easily accessible health information regarding reduction of risk factors for CVD, both online and within hospital outpatient clinics, is limited to smoking cessation, reducing alcohol intake, improving diet, and becoming physically active [25, 44–48]. Unless specifically searched for, the importance of good oral hygiene for cardiovascular health is rarely mentioned.

Compounding this lack of awareness is that many oral health intervention studies conducted within hospital wards, clinics, or outpatient facilities, focus on the education of clinical staff [49], namely nurses [50–53], and do not empower patients. Interventions within a cardiac rehabilitation out-patient facility have only involved self-reported oral health knowledge and behaviour [54, 55]. Currently, no intervention study has reported on the impact of oral health education on objective oral health outcomes for patients with CVD.

In conjunction with traditional methods of patient education, the use of digital media has been proven an effective strategy to improve patient’s knowledge and confidence regarding CVD [56, 57], even in individuals with low literacy levels [58, 59]. Additionally, digital media based education strategies in the dental clinic waiting room have been shown to improve self-reported oral hygiene habits [60]. To the best of our knowledge, there are no published studies seeking to improve oral health knowledge, motivation, and confidence by using a digital education approach in a cardiac rehabilitation setting. The aim of this study is to assess whether empowering patients by incorporating individualised OHI partnered with a digital oral health education (DOHE) can improve the oral health of CVD patients in cardiac rehabilitation out-patient facilities.

## Methods

### Study design

**Fig 1:**
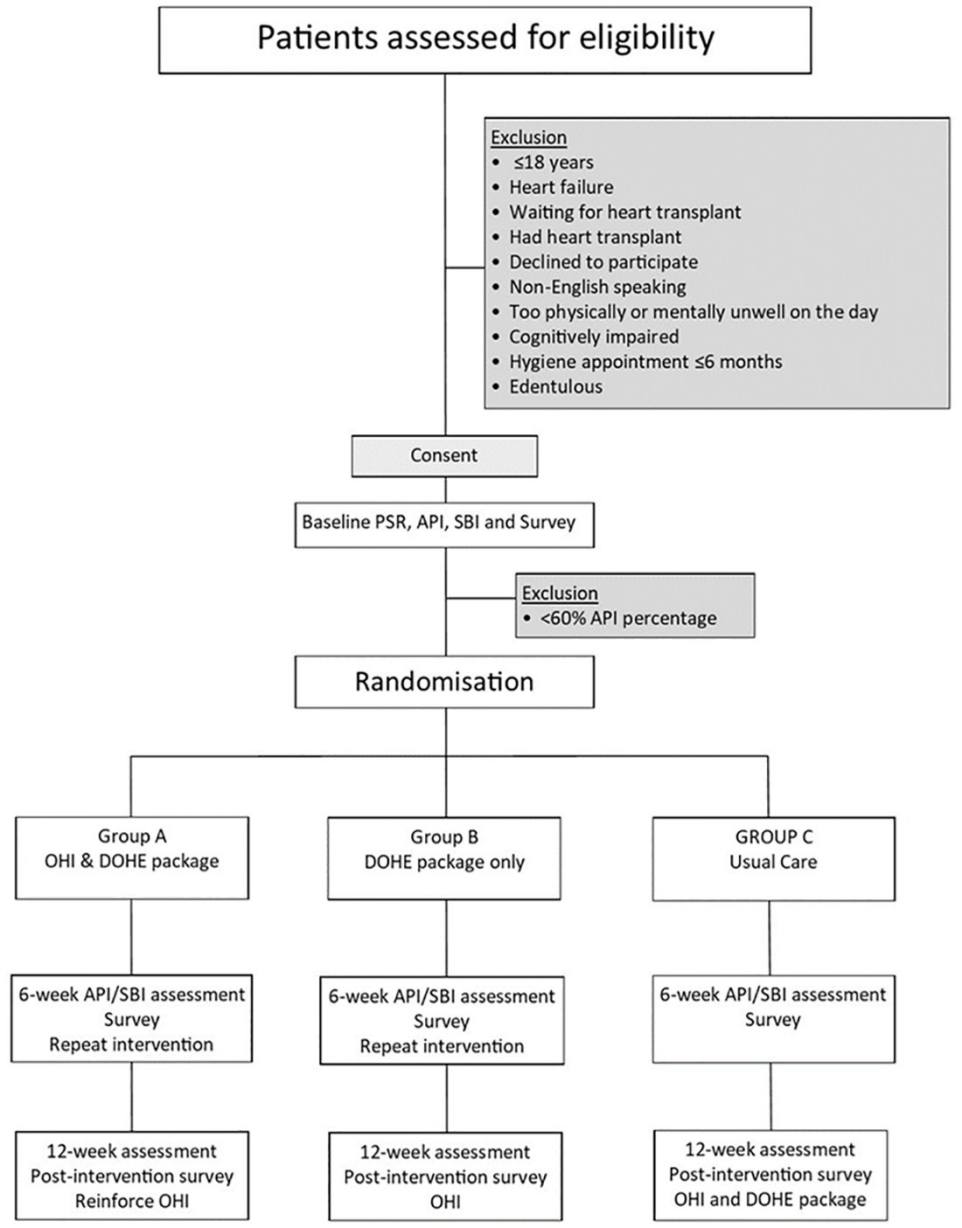
Study flow diagram.

A dual-center, single-blind, parallel design RCT of 165 patients attending an outpatient cardiac rehabilitation center within two public hospitals in Sydney, Australia. See Fig 1 for study flow diagram. The intervention populations will receive either: personalised OHI partnered with DOHE which will comprise a tablet delivered specifically selected series of advertisement free, web-based publicly available educational videos covering oral hygiene instruction and the link between CVD and oral health; or the DOHE alone. The control population will receive usual care/no oral health education as detailed below. The examiner will be blinded to the group allocation.

### Patient population

The study population will be patients presenting to the outpatient cardiology rehabilitation centre participating in the Cardiac Education and Assessment Program (CEAP) at Westmead and Blacktown Mt Druitt public hospitals. Patients attend CEAP for a range of heart conditions such as coronary heart disease, heart failure, and those waiting for, or who have had a heart transplant. Due to the variety of heart conditions and co-morbidities, patients are placed on either a 6-week or 12-week program and can attend once or twice weekly. The program provides personalised support, exercise and education to help strengthen the heart after a cardiovascular event and helps to lower the risk of future cardiovascular event [61]. However currently, the program does not include information regarding oral health for good cardiovascular health.

### Participant eligibility

**Fig 2:**
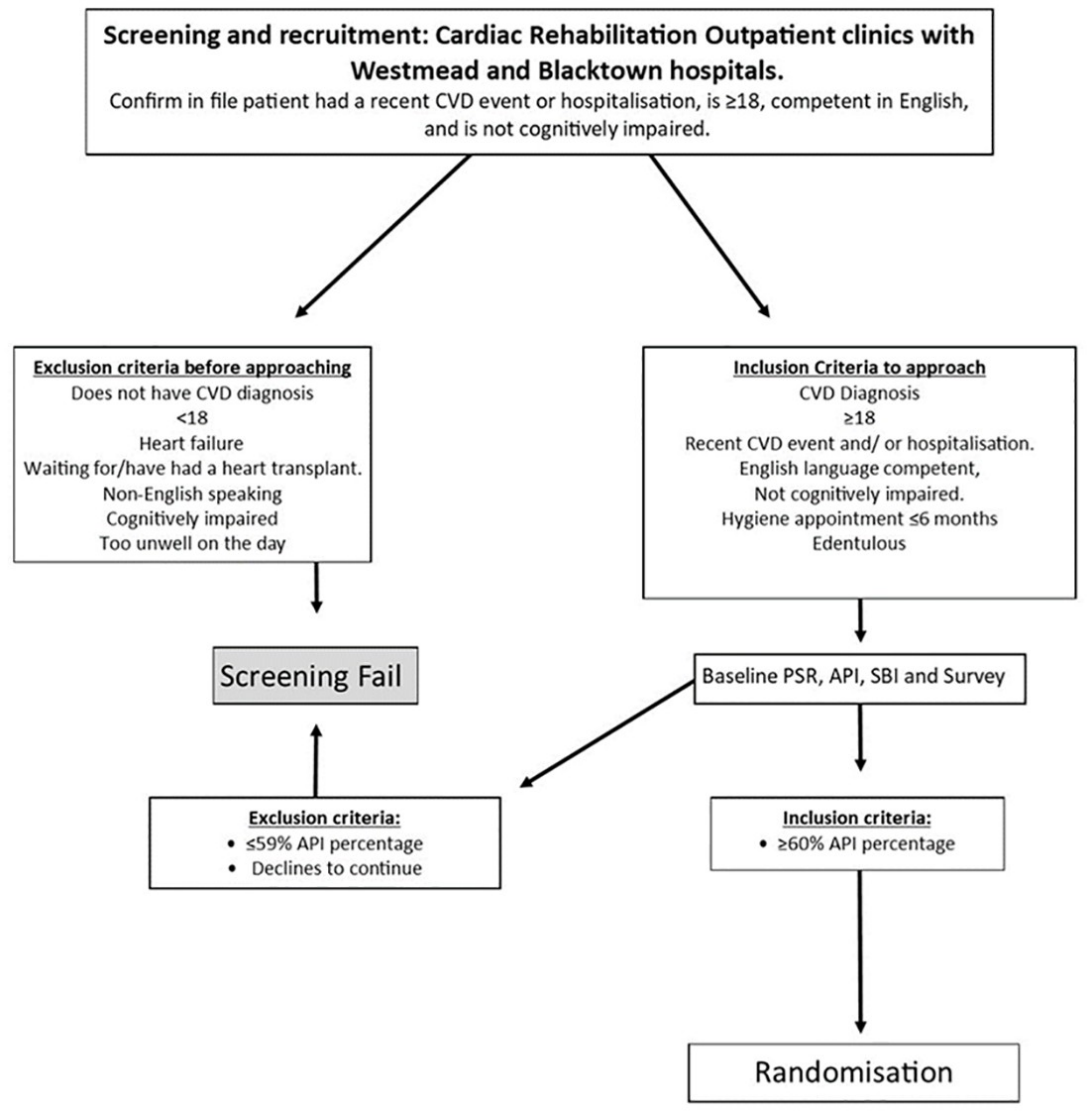
Patient recruitment flow diagram.

A clinical examination comprising of periodontal screening and recording (PSR) [62], sulcus bleeding index (SBI) [63], and approximal plaque index (API) [64], followed by a self-report questionnaire will be used to screen participants. Inclusion criteria are patients 18 years or older with a recent diagnosis of CVD, recent myocardial infarction and/or recent hospitalisation resulting from CVD, API score of equal to or greater than 60% and those with competent English language skills. Exclusion criteria include: patients with limited English language skills to provide informed consent, a diagnosis of heart failure or who have/are waiting for a heart transplant, are too physically or mentally unwell on the day or have a cognitive impairment, have visited an oral health practitioner for treatment of periodontal disease (supra- and/or subgingival scaling and root planning) in the last 6 months, have an API score of less than 60%, or are edentulous. A summary of the recruitment process is provided in Fig 2.

### Recruitment and consent

One hundred and sixty-five participants will be recruited from the Westmead and Blacktown Mt Druitt CEAP clinics either before or after their CEAP session. A member of the research team will explain the study to potential participants. Electronic consent forms will be provided on iPads, hosted on the Western Sydney Local Health District (WSLHD) Research Electronic Data Capture (REDCap) platform [65]. The participant must provide consent before being able to progress to the data collection stage. Recruitment for this study commenced August 22^nd^, 2023.

### Randomisation

Participants will be randomised into a 1:1:1 ratio into three arms of the study, stratified by age (≤64 years or ≥65 years) and clinic site (Westmead or Blacktown Mt Druitt). A randomisation allocation table will be computer generated and REDCap will be used for the allocation of participants to each group. Due to the nature of the interventions within the trial, participants cannot be blinded.

### Interventions

Participants will be randomised into one of 3 arms as follows: individualised OHI partnered with a DOHE package (Group A), DOHE package only (Group B), and control/usual care (Group C). Each intervention will be repeated at the 6-week follow up. At the end of their final follow up (12-weeks), Group B and Group C will receive OHI. Group C will also be shown the DOHE package. A single trained and calibrated oral health therapist (OHT), blinded to the treatment allocations will perform the baseline and follow up assessments. After the initial assessment of participants for eligibility, if any obvious dental issues (without the use of radiographs) are detected, patients will be provided with a letter to their oral health practitioner for further investigation.

### Oral hygiene instruction

Ensuring basic OHI are the same, an evidence based, peer-reviewed oral hygiene standard operating procedure was created to calibrate research assistants, OHT and/or dental students who will be delivering the tailored oral health instructions. See S1 File. Individualised instructions will be based on participants’ API and SBI assessment results as this will show the assistant where the participant is missing, as well as the current oral hygiene items the participant uses. Introduction of interdental cleaning aids may be suggested, however no oral hygiene items will be issued to participants.

### DOHE

Videos to be included in the digital oral health education package will be evaluated and rated by a multi-disciplinary panel to ensure content validity and consumer engagement. Included videos are publicly available and advertisement free; created by either government bodies, or relevant health bodies such as the Australian Dental Association. Educational messages will involve the link between oral health and CVD, as well as oral hygiene practices [57]. These video bases OHI will mirror instructions given face-to-face. If in the appropriate arm, these will be delivered at baseline after the self-report form has been completed.

## Study outcomes

### Primary outcome

The primary outcome of the study will be whether a greater proportion of Group A will decrease their API score compared group C, evaluated at 6 weeks.

### Secondary outcomes

The secondary outcomes are:

- A greater proportion of Group A maintain or further reduce API score, compared to Group C, at 12 weeks.
- A greater proportion of Group B will have greater success at reducing their API score, compared to Group C at both 6- and 12-week follow up. achieved at the 6-week follow-up at the 12-week follow up.
- Mean improvement of API & SBI percentages (all groups).
- Proportion of participant achieving a clinically significant reduction in API and/or SBI scores to ≤35% and ≤25% respectively.
- Improved oral hygiene practices–an increase in brushing frequency and/or inclusion of interproximal cleaning, from baseline.
- Motivation and confidence to perform oral hygiene–yes/no.
- Motivation to see an oral health practitioner regularly–yes/no.
- Knowledge of the link between heart health and oral health–yes/no.
- Patient perceptions of a nurse’s role in their oral health care–yes/no.

## Data collection

To assess short- and long-term changes to oral hygiene habits, data will be collected at baseline, and the 6-and 12-week follow ups. Data will be stored on REDCap. Baseline data will include demographic information, medical history, weight (in kg), height (in cm), body-mass index (BMI), oral hygiene practices, and oral health knowledge associated with CVD. Weight, height, and BMI are recorded when patients first attend CEAP, therefore this information will be retrieved from the patients’ medical record. Questionnaires will be hosted on REDCap accessed using iPads (S2 File). Clinical parameters recorded at baseline, the 6- and 12-week follow-up, will be completed using a disposable World Health Organisation (WHO) periodontal probe and mirror, with tricolour disclosing gel. For timeline and mode of collection see Table 1.

**Table 1:** Timing and mode of collection of study data.

Clinical measurements and Surveys	Baseline	6-week Assessment	12-week Final Assessment
Demographic information and medical history and behaviour/risk factors	X		
Clinical measurements PSR	X		
Clinical measurements PSR/API/SBI	X	X	X
Oral health Perceptions and knowledge	X	X	X
Oral hygiene habits	X	X	X
Motivation and confidence and to improve oral hygiene practices	X	X	X
Perceptions of nurses in oral health care	X		
Do they have an oral health practitioner and see them regularly	X		
Motivation to see oral health practitioner	X	X	X

## Data management

Identifiable data will be housed on secure servers within the WSLHD network. The server is managed and maintained by WSLHD Digital Health Services. Each patient will be assigned a code–the patient’s name and contact details will be linked to the study code and stored in an encrypted and password protected folder, separate to all other data files, with access to this folder limited to the principal investigator and coordinating principal investigator. All other data files will contain only the study code. Participant consent forms will be in digital form stored on REDCap, refer to Appendix SI 3. Data sheets will contain the study code, age, and sex, and will be stored electronically on REDCap. De-identified data will be used for any analyses and publications and will be retained for a period of 5 years following the study completion. We anticipate no harm resulting from the intervention, as such we do not require a data safety monitoring board.

## Clinical measurements

Periodontal health will be assessed based on the SBI, API, and PSR [62–64]. Firstly, to assess the SBI a WHO periodontal probe is gently inserted into the interproximal areas of the buccal sulcus of the first and fourth quadrant, and palatal/lingual sulcus surfaces of the second and third quadrant. Any evidence of bleeding on probing (BOP) is recorded [63]. Next, the API is completed by using a micro brush to place a small amount of the disclosing liquid on and around the interdental papillae on the buccal surfaces of the teeth in the second and third quadrants, and the lingual/palatal surfaces of the teeth in the first and fourth quadrants. After allowing the patient to rinse, interproximal sites with any remaining disclosing solution are recorded as a positive reading [64]. The number of positive readings are expressed as a percentage of the total readings. See Table 2 for API/SBI classifications.

**Table 2:** Adapted API/SBI clinical measurement results [66].

	API	SBI
**Poor oral hygiene**	>35%	>25%
**Fair oral hygiene**	35%	15–25%
**Good oral hygiene**	<25%	<15%

Finally, the PSR is completed by dividing the mouth into sextants and, starting in sextant one, gently inserting a WHO probe into the gingival sulcus taking a 6-point measurement of each tooth. Specific markings on the probe relate to a measurement, indicating which code to classify the sextant as [62]. See Table 3 for code definitions. Any participants returning a PSR code of 3 or 4 will still be included in the study, however, will be provided with a referral letter and strongly advised to seek follow-up with an oral health practitioner for further assessment.

**Table 3:** Adapted PSR code definitions [62, 67].

Code	Clinical Signs
**0**	Absence of clinical signs: no calculus, or BOP Coloured band on WHO probe completely visible.
**1**	No calculus. BOP Coloured band on WHO probe completely visible.
**2**	Supra and/or subgingival calculus BOP Coloured band on WHO probe completely visible.
**3**	Periodontal pocket 3.5mm-5.5mm Coloured band on WHO probe partially visible
**4**	Periodontal pocket >5.5mm deep Coloured band on WHO probe no longer visible
**X**	Sextant absent of dentition
*	Periodontal abnormalities which include: Furcation involvement Mobility Mucogingival problems for example: exudate, severe oedema >3.5mm recession The * is recorded next to the sextant number code. E.g. “3*”

## Sample size

The required sample size for this study is a total of 165 (1:1:1 Group A: Group B: Group C). This calculation includes provision for a 10% attrition rate, two tailed tests, type 1 error of 5%, and will have 80% power to detect any significant difference between Group A and C. This calculation is based from an API score reduction of 25% in the intervention arm of Ziebolz, et al 2009 study [68]. Due to the current study’s exclusion criteria of ≤59% API score, it is expected the proportion of Group A lowering their API score will be ≥40% and Group B will be ≥30%, compared to only 15% in Group C. However, the sample size is only powered to detect a difference in percentage proportion of those between Group A and C, and has poor power to detect a difference between Group B and C.

## Statistical analysis

The statistical plan for this study will be determined prior to study completion, where all relevant assumptions will be checked. Analysis of the difference in API will be according to the intention-to-treat principle where participants are analysed in the arm they have been allocated. The level of statistical significance will be set at p-value <0.05. The primary analysis will be a logistic regression analysis to assess differences between groups for API scores between baseline and follow-up. Covariates used for adjusted analyses will include treatment group, age, and CEAP location. Subgroup analysis of the intervention effect sex, ethnicity, daily brushing habits, and daily interdental cleaning habits. The long-term effects of the treatments will be assessed using a mixed logistic regression with a random effect for patient, and a fixed effect for treatment, and a time and treatment interaction will be added to assess the treatment effect at each time point. Other outcome measures will be reported as descriptive statistics. If missingness for the primary outcome variable is high, missingness at random will be assessed.

## Process evaluation

The effectiveness of the intervention will be evaluated with self-assessments of the educational value and tolerability of the study incorporated into follow-up questionnaires. Additionally, a member of the research team will contact participants via telephone at study completion, obtaining their perception of the study intervention. An anonymous questionnaire, with implied consent by clicking the link will also be given to the nursing staff to assess their oral health related knowledge, perception of the intervention’s importance and effectiveness, and ways it may be better incorporated into CEAP–see Appendix SI 4. As no relevant nursing questionnaire exists to determine the outcomes of this study an adapted questionnaire was developed from previously established tools [69–72]. A recruitment log will record patients who do not wish to participate, or for those who are ineligible.

## Conclusion

The association between periodontitis and CVD has been established [18, 21, 29]. For most patients with CVD, the accessibility of information regarding the association between periodontitis and heart disease and the impacts oral hygiene practices have on their cardiovascular health, are almost non-existent [25, 44–48]. As such, oral health knowledge and the importance of oral hygiene in this population is low [54]. Empowering patients with individualised OHI is known to improve oral health [30], and digital educational programs have proved effective in increasing knowledge, confidence, and motivation to improve health outcomes [57], even when literacy levels are low [58, 59]. Therefore, this study will assess if the introduction of individualised OHI partnered with a DOHE instrument will improve oral hygiene practices, knowledge, and motivation to improve the oral health of patients with CVD.

## Ethics

Ethics approval was sought from the Western Sydney Local Health District Human Research Ethics Committee: 2023/ETH00516. During recruitment, baseline and follow-up assessments, participants will be informed of their right to decline to take part or withdraw from the trial at any time. It will be highlighted that non-participation or withdrawal will in no way affect their current or future care at the hospital. The authors will seek approval of any variations required to be made to the protocol. Informed consent will be gained from all participants before being included in the study. The trial registration number is ACTRN12623000449639p and has been registered prospectively (02/05/2023) with the Australian and New Zealand Clinical Trials Registry (ANZCTR).

## Strengths and limitations of this study

- This is the first study to implement digitally delivered oral health education, as well as personalised oral hygiene instruction to patients with cardiovascular disease, within a cardiac rehabilitation outpatient clinic.
- This study will test the impact of empowering patients with these educational strategies within cardiac rehabilitation clinics.
- The design of this pilot study indicates that findings cannot be used to make conclusions about the effectiveness of the interventions on reducing systemic inflammation.
- The results may not relate to more diverse communities as participants are excluded if they have limited English.
- Due to the nature of the clinical intervention, it is not possible to blind those delivering or receiving the intervention.

## Dissemination

The results from this study will be published in peer reviewed journals and presented nationally and internationally at both oral health and cardiology conferences.

## Supporting information

S1 File Oral hygiene instruction standard operating procedure. (DOCX)

S2 File Patient questionnaire. (PDF)

S3 File Patient consent form. (DOCX)

S4 File Nursing staff questionnaire. (PDF)

S5 File Spirit checklist. (PDF)

S1 Protocol (DOCX)
