# Supplementary material for: Evaluating the impact of oral hygiene instruction and digital oral health education within cardiac rehabilitation clinics: A protocol for a novel, dual centre, parallel randomised controlled trial
Source: PLoS One. 2024 Jul 11;19(7):e0306882. doi: 10.1371/journal.pone.0306882 (PMC11239009; doi:10.1371/journal.pone.0306882)
Supplement: S1 File — (DOCX) [file pone.0306882.s001.docx]

**Oral Hygiene Instruction - Standard Operating Procedure (SOP)**

Initial instructions:

A toothbrushing and interdental cleaning routine should occur at least twice daily [1], preferably with interdental brushes [2] and a powered oscillating toothbrush [3]; once in the morning and once at night just before bed. The toothbrushing component should last for at least 2 minutes [4].

**Interdental Cleaning**

Instructions for interdental brushing*

1. Wet bristles of interdental brush
2. Insert into the interproximal area at the gingival margin.
3. Gently move the brush in and out in the same space at least 5 times before removing.
4. Start at the interproximal area of the most posterior teeth on the right-hand side of the maxilla, moving across until the last interproximal area of the left-hand side had been completed.
5. Repeat process for all mandibular interproximal areas.
6. Rinse all debris from the interdental brush away and allow it to air dry.
7. After interdental brushing is complete, move to toothbrushing [5].

Instructions for string-floss or flossette**

1. Remove a 30cm piece of floss from the packet. If using flossette go to step c.
2. Start at one end of the floss. It can be held between thumb and index finger or between thumbs for maxillary dentition, holding between two index fingers for mandibular dentition.
3. Move the floss slowly between the teeth using a small horizontal sawing motion with slight pressure.
4. Once in the interproximal space, curve the floss around one tooth in a C shape.
5. Manoeuvre the floss so it is slightly under the gingival margin, then gently pull the floss out of the space, keeping the C shape on the tooth.
6. Move to the next bit of floss, ensuring the used part is not going back into the interproximal space.
7. If using flossette, wipe or rinse floss between each new interproximal space.
8. Enter the same space, however, curve the floss around the neighbouring tooth and repeat step d-f.
9. Start at the distal surface of the most posterior tooth on the right-hand side and repeat steps until all maxillary and then mandibular teeth have been flossed.
10. If using flossette, give a final rinse at the end and allow to air dry. Replace when floss snaps.
11. After flossing is complete, brush teeth [5].

**Toothbrushing**

Instructions for manual toothbrushing - modified bass technique:

1. Place a pea sized amount of toothpaste on a soft bristle toothbrush.
2. Angle the toothbrush at a 45-degree angle to the gingival margin.
3. Brush approximately two teeth at a time in a small circular movement feeling for a slight massage of the gums.
4. Start on the buccal surface at the most posterior tooth on the upper right-hand side.
5. Complete 6 or 7 small circles along the gingival margin and then flick the toothbrush away twice then move slowly over each tooth until reaching the most posterior tooth on the left-hand side.
6. Move the toothbrush head to palatal surface of the most posterior tooth on the left-hand side and slowly move across the teeth until the toothbrush is back to the first tooth.
7. Repeat steps D-F for mandibular teeth.
8. Brush biting surfaces last and in a sawing motion, approximately 10 times per quadrant starting at the back of the most posterior tooth moving towards the anterior. Repeat in all relevant quadrants.
9. Spit out excess toothpaste, do not rinse [6,7].

Instructions for toothbrushing using an electric/powered toothbrush with an oscillating head:

1. Place a pea sized amount of toothpaste on a soft bristle toothbrush head.
2. Place the toothbrush at a 45-degree angle on the buccal surface of the most posterior tooth on the upper right-hand side and turn on.
3. Adapt the toothbrush around the gingival margin of each tooth whilst slowly moving over each tooth towards the most posterior tooth on the left-hand side.
4. Move the toothbrush head to palatal surface of the most posterior tooth on the left-hand side and slowly move across the teeth until the toothbrush is back to the first tooth.
5. Repeat steps C and D for mandibular teeth.
6. Once finished brushing the buccal and lingual/palatal surfaces of the teeth, adapt the toothbrush head to the biting surfaces of the teeth.
7. Start at the very back of the last tooth in the quadrant and moving slowly from the back to the anterior. Repeat in all relevant quadrants.
8. Spit out excess toothpaste, do not rinse [7].

* Advise the patient these can be used for between 2-3 weeks or until the bristles start to thin. When this occurs, use a new interdental brush. **Interdental brushes are the preferred method of interproximal cleaning and should be prioritised. Flossing instruction should only be used in the instance where an interdental brush will not fit in the interdental space.

**References**

[1] T. Attin and E. Hornecker, “Tooth brushing and oral health: how frequently and when should tooth brushing be performed?,” *Oral Health Prev. Dent.*, vol. 3, no. 3, pp. 135–140, 2005.

[2] H. Worthington *et al.*, “Home use of interdental cleaning devices, in addition to toothbrushing, for preventing and controlling periodontal diseases and dental caries,” *Cochrane Database Syst. Rev.*, no. 4, 2019, doi: 10.1002/14651858.CD012018.pub2.

[3] M. Yaacob *et al.*, “Powered versus manual toothbrushing for oral health,” *Cochrane Database Syst. Rev.*, no. 6, 2014, doi: 10.1002/14651858.CD002281.pub3.

[4] A. Gallagher *et al.*, “The Effect of Brushing Time and Dentifrice on Dental Plaque Removal in vivo,” vol. 83, no. 3, p. 6, 2009.

[5] E. Wilkins and D. Lyle, “Interdental Care and Irrigation,” in *Clinical Practice of the Dental Hygienist*, 11th ed., Philadelphia: Lippincott Williams & Wilkins, 2013, pp. 408–422.

[6] M. Poyato-Ferrera, J. Segura-Egea, and P. Bullón-Fernández, “Comparison of modified Bass technique with normal toothbrushing practices for efficacy in supragingival plaque removal: Modified Bass versus normal toothbrushing,” *Int. J. Dent. Hyg.*, vol. 1, no. 2, pp. 110–114, May 2003, doi: 10.1034/j.1601-5037.2003.00018.x.

[7] C. Wyche, “Indices and Scoring Methods,” in *Clinical Practice of the Dental Hygienist*, 11th ed., E. Wilkins, Ed., Philadelphia: Lippincott Williams & Wilkins, 2013, pp. 311–335.
