# Supplementary material for: Evaluating the impact of oral hygiene instruction and digital oral health education within cardiac rehabilitation clinics: A protocol for a novel, dual centre, parallel randomised controlled trial
Source: PLoS One. 2024 Jul 11;19(7):e0306882. doi: 10.1371/journal.pone.0306882 (PMC11239009; doi:10.1371/journal.pone.0306882)
Supplement: S2 File — (PDF) [file pone.0306882.s002.pdf]

Clinical Measurements BL

Please complete the survey below.

Baseline: Physical & Anthropometric Measures

Date of Baseline Assessment

____

Assessment location

◯ Westmead CEAP
◯ Blacktown CEAP

Sex

◯ Male
◯ Female

DOB

____

Age in years

____

Age Group

____

Age Group

◯ 64 and under
◯ 65 and over

Do you identify as Aboriginal or Torres Strait Islander?

◯ Aboriginal
◯ Torres Strait Islander
◯ Both Aboriginal and Torres Straight Islander
◯ Neither Aboriginal or Torres Straight Islander

What is your ethnic background:

☐ Australian
☐ New Zealander
☐ Asian
☐ Indian
☐ Middle Eastern
☐ European
☐ North American
☐ South American
☐ African
☐ Decline to answer
☐ Other, ______

Employment status

◯ Full time
◯ Part time
◯ Unemployed
◯ Retired

What is your household yearly income?

◯ < $25, 000
◯ $25, 001 - $49,000
◯ $49,001 - $80, 000
◯ $80,001 - $120, 000
◯ $120, 001 - $180, 000
◯ $180, 001+
◯ Prefer not to say

What is your highest educational qualification?	◯ No formal schooling<br>◯ Primary school<br>◯ Secondary school<br>◯ TAFE<br>◯ College or University<br>◯ Post grad<br>◯ Prefer not to say
Do you have:	☐ Private health insurance<br>☐ Pensioner concession card<br>☐ Health care card<br>☐ Department of Veteran Affairs (gold) card<br>☐ None of the above
What cardiovascular condition do you have?	☐ Atherosclerosis/ Coronary heart disease/Ischemic heart disease<br>☐ Atrial fibrillation<br>☐ Aneurysms<br>☐ Angina<br>☐ Aortic condition<br>☐ Arrhythmia<br>☐ Bradycardia<br>☐ Endocarditis<br>☐ Pericarditis<br>☐ Pulmonary embolism<br>☐ Pacemaker<br>☐ Rhematic fever<br>☐ Tachycardia<br>☐ Valve replacement<br>☐ Other. ______
What other medical conditions do you have?	☐ None<br>☐ Type 1 Diabetes<br>☐ Type 2 Diabetes<br>☐ Hypertension (High blood pressure)<br>☐ Chronic kidney disease<br>☐ Rheumatoid arthritis<br>☐ Anxiety<br>☐ Depression<br>☐ Other. ______
How often do you drink alcohol:	◯ Never<br>◯ Seldom<br>◯ Monthly<br>◯ Weekly<br>◯ 1-2 daily<br>◯ 3-4 daily<br>◯ 5-6 daily<br>◯ >6 daily
Smoking status	◯ Current smoker cigarettes<br>◯ Current smoker e-cigarettes/vaping<br>◯ Ex-smoker<br>◯ Non-smoker<br>◯ In the process of quitting
How often do you smoke?	◯ Daily<br>◯ Socially/Occasionally

How long after waking do you have your first cigarette?	◯ 0-5 mins<br>◯ 6-30 mins<br>◯ 31-60 mins<br>◯ 60+ mins
Are you interested in quitting?	◯ Yes<br>◯ No
Would you like a referral to the Quitline?	◯ Yes<br>◯ No

Oral Hygiene Habits

How often do you visit a dental clinic?	◯ 3 monthly<br>◯ 6 monthly<br>◯ Yearly<br>◯ Irregular<br>◯ For pain/emergency appointments only
Why?	☐ Cost<br>☐ Can't get to the dentist easily<br>☐ It is not a priority<br>☐ Too busy to see a dentist<br>☐ Public dentistry wait times<br>☐ Dental anxiety<br>☐ Other (please specify): ______
Teeth and gum health is important to me.	◯ Yes<br>◯ No
Why?	______________________________
How often do you brush your teeth and/or dentures?	◯ Never<br>◯ A few times a week<br>◯ 1 x daily<br>◯ 2 x daily<br>◯ More than twice a day<br>◯ After every meal
What type of tooth brush do you use?	☐ None<br>☐ Soft bristle manual<br>☐ Medium bristle manual<br>☐ Electric toothbrush with oscilating head<br>☐ Electric toothbrush non-osiclating head
Do you clean in between your teeth?	☐ No<br>☐ Yes with floss/flossettes<br>☐ Yes with an interdental Brush<br>☐ Yes with a water Flosser<br>☐ Yes with a tooth pick

When do you clean in between your teeth?	☐ After every meal<br>☐ 2 x daily<br>☐ 1 x daily<br>☐ Irregular<br>☐ When food is stuck in between teeth<br>☐ Never
Do you use mouthwash or rinse with water?	☐ Never<br>☐ After brushing<br>☐ After meals<br>☐ After a cigarette/e-cigarette<br>☐ Irregular
What do you use as mouthwash?	◯ Regular rine such as listerine or Plax<br>◯ Savacol<br>◯ Salt water<br>◯ Plain water
What type of tooth paste do you use?	☐ Toothpaste with fluoride<br>☐ Toothpaste without fluoride<br>☐ Unsure

Anthropotic measures

Weight in kgs	__________________________________
Height in cms	__________________________________
BMI kg/m2	__________________________________
Heart rate	__________________________________
Systolic blood pressure mmHg	__________________________________
Diastolic blood pressure mmHg	__________________________________
Number of teeth	__________________________________
Highest PSR Code	◯ 1<br>◯ 2<br>◯ 3<br>◯ 4
API %	__________________________________
API Calc	__________________________________

API Group	◯ 59 and under<br>◯ 60 and over
SBI %	______________________
Issued dental letter	◯ Yes<br>◯ No
For:	☐ Obvious caries (open cavity)<br>☐ Suspected caries (shadowing/haloing)<br>☐ PSR 3-4<br>☐ Exudate upon palpation<br>☐ Abscess<br>☐ Pain<br>☐ Other ______
