# Supplementary material for: Evaluating the impact of oral hygiene instruction and digital oral health education within cardiac rehabilitation clinics: A protocol for a novel, dual centre, parallel randomised controlled trial
Source: PLoS One. 2024 Jul 11;19(7):e0306882. doi: 10.1371/journal.pone.0306882 (PMC11239009; doi:10.1371/journal.pone.0306882)
Supplement: S3 File — (DOCX) [file pone.0306882.s003.docx]

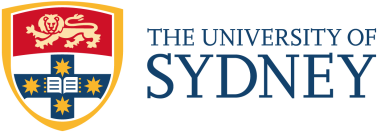

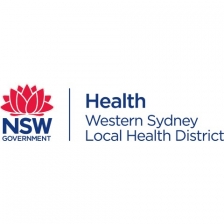


**Participant Information Sheet/Consent Form**

**Interventional Study** - *Adult providing own consent*

| **Title** | Oral hygiene instruction and digital oral health education within cardiac rehabilitation clinics, a novel approach to improving oral and cardiovascular health. |
| --- | --- |
| **Short Title** | Health impacts on the heart, the mouth is where it starts |
| **Principal Investigator** | Professor Clara Chow |
| **Location** | Westmead and Blacktown Cardiac Rehabilitation Facilities. |

**Part 1 What does my participation involve?**

**1 Introduction**

You are invited to take part in this research project. This is because you have heart disease. The research project is testing a whether toothbrushing instruction and educational videos improves your teeth and gum health and your knowledge of its impacts on heart disease.

This Participant Information Sheet/Consent Form tells you about the research project. It explains the tests and treatments involved. Knowing what is involved will help you decide if you want to take part in the research.

Please read this information carefully. Ask questions about anything that you don’t understand or want to know more about. Before deciding whether or not to take part, you might want to talk about it with a relative, friend or your local doctor.

Participation in this research is voluntary. If you don’t wish to take part, you don’t have to. You will receive the best possible care whether or not you take part.

If you decide you want to take part in the research project, you will be asked to sign the consent section. By signing it you are telling us that you:

• Understand what you have read

• Consent to take part in the research project

• Consent to have the tests and treatments that are described

• Consent to the use of your personal and health information as described.

You will be given a copy of this Participant Information and Consent Form to keep.

**2 What is the purpose of this research?**

Health professionals have long established a link between gum disease and heart disease. However, mouth health awareness and its importance on heart health is low within the community. Therefore, the purpose of this research is to increase this awareness and improve mouth health status via education videos and/or toothbrushing instruction.

The results of this research will be used by a member of the research team (Ms Lauren Church) to obtain a Doctor of Philosophy (Dentistry) degree.

This research has been initiated by the study doctors Professor Clara Chow and Professor Axel Spahr and is being conducted by The Western Sydney Local Health District.

**3 What does participation in this research involve?**

You will be participating in a randomised controlled research project. Sometimes we do not know which treatment is best for treating a condition. To find out we need to compare different treatments. We put people into groups and give each group a different treatment. The results are compared to see if one is better. To try to make sure the groups are the same, each participant is put into a group by chance (random). You will be placed into one of three groups at random. The research team and you do not have control over which group you will be put into. The groups are:

Group A) Personalised toothbrushing instruction and watching mouth and heart health videos.

Group B) Watching mouth and heart health videos only.

Group C) Usual care. – Meaning you will not have mouth health education until the end of the study.

This study will be conducted over a 12-week period within the cardiac rehab facility. No matter which group you a put in, you will need to attend three visits. At each visit you will be taken into a private room either before or after your cardiac rehab appointment to have simple gum measurements taken, complete a short survey, and depending on the group you were placed in, to have your groups education repeated.

The gum measurements will require you to lay in a chair with your mouth open. A gum measurement tool will be used to assess the health of your gums and to see if you bleed easily when your gums are touched by the tool. The tool is placed gently into your gum line to see how deep the tool goes.

A micro-brush will also be used in your mouth to apply a gel in between your teeth. After it is placed you will be asked to rinse with water to see if there is any colour remaining on your teeth The colour of the gel is purple and can change to pink and blue depending on the bacteria you have on your teeth and gums and is easily removed when you next eat or brush your teeth. Vasoline will be placed on your lips to make sure you do not get any gel on your lips.

The surveys will be electronic via an iPad provided to you by the research team. There are videos which are about mouth care and heart health. They are between 1 – 2 minutes long and have been selected by health professionals. You will watch in private on the same iPad provided to you in the same room you have your gum measurements.

As the first visit requires more questions than the others, this visit may take approx. 30-45 minutes.

The second visit, which will occur 6 weeks after your first visit should take approx. 15mins.

The last visit, which will happen at 12 weeks after your first visit may take up to 30 minutes if you were in group B or group C. This is because at the final visit you will be given personal tooth brushing instruction and will be able watch the videos.

At the end of the study, a member of the research team will contact you via telephone to discuss your opinion of the study. In addition, the researchers would like to have access to your medical record to obtain information relevant to the study.

This research project has been designed to make sure the researchers interpret the results in a fair and appropriate way and avoids study doctors or participants jumping to conclusions.

There are no additional costs associated with participating in this research project, nor will you be paid. All videos will be shown to you free of charge.

**4 What do I have to do?**

As a participant in this study, you will be required to have gum measurements taken and complete a survey at each visit. These visit will be completed on the same day either before or after your rehab appointment not requiring you to come in for a separate visit. However, if your rehab appointments finish before the end of the study, you will still need to come in for your 6 and/or 12 week follow up visit.

| Measurements and Surveys | Baseline/ 1^st^ visit | 6-week  Visit | 12-week  Final visit | End of study |
| --- | --- | --- | --- | --- |
| Information about you, such as medical history, address, working status etc. | X |  |  |  |
| Gum measurements | X | X | X |  |
| Mouth health survey | X | X | X |  |
| Follow up phone call |  |  |  | X |

**5 Other relevant information about the research project**

There will be a total of 165 participants in this study, allocated to one of three arms (55 participants per arm). The project involves researchers from Westmead Hospital, and the Westmead Centre for Oral Health.

**6 Do I have to take part in this research project?**

Participation in any research project is voluntary. If you do not wish to take part, you do not have to. If you decide to take part and later change your mind, you are free to withdraw from the project at any stage.

**7 What are the alternatives to participation?**

You do not have to take part in this research project to receive treatment at this hospital. If you are interested in learning more about your mouth health and how it can impact your heart health, you can discuss this with a dentist or local doctor.

**8 What are the possible benefits of taking part?**

We cannot guarantee or promise that you will receive any major benefits from this research. The personalised toothbrushing instruction and educational videos may improve your mouth health and increase your knowledge on mouth health and its impact on heart health. However, the possible benefit may include identification of any teeth or gum issues early, with a letter you can take to your local dentist and have any treatment required early.

**9 What are the possible risks and disadvantages of taking part?**

The gum measurements taken may cause some discomfort or minor bleeding which typically stops within 10-20 seconds. There are no known risks of watching videos on mouth health and heart disease. It will extend your time spent in the cardiac rehab by approximately 30-45 minutes. You will be required to return for your 6-and 12-week assessment if your cardiac rehabilitation has finished.

**10 Can I have other treatments during this research project?**

Whilst you are participating in this research project, it is recommended you postpone any dental appointments involving a scale and clean for the duration of the study. However, you can attend any other dental appointments if needed and when your 12-week visit has been finished, you can go to your dentist for a scale and clean. You can continue with any medications and/or treatments you are required to take for any medical conditions.

**11 What if I withdraw from this research project?**

If you decide to withdraw from the project, please notify a member of the research team before you withdraw. If you do withdraw your consent during the research project, the study doctor and relevant study staff will not collect additional personal information from you, although personal information already collected will be retained to ensure that the results of the research project can be measured properly and to comply with law. You should be aware that data collected by the researcher up to the time you withdraw will form part of the research project results. If you do not want them to do this, you must tell them before you join the research project.

**12 Could this research project be stopped unexpectedly?**

It is not anticipated that this project will be stopped for any reason.

**13 What happens when the research project ends?**

We will report the results of the study in peer-reviewed papers and publish reports on our institutional websites. A summary of the results of the study will be sent to you in approximately 2025.

**Part 2 How is the research project being conducted?**

**14 What will happen to information about me?**

By signing the consent form you consent to the study doctor and relevant research staff collecting and using personal information about you for the research project. Any information obtained in connection with this research project that can identify you will remain confidential. The information will only be accessible to the study investigators and will be kept in a locked filing cabinet at the Westmead Applied Research Centre. A source document will be generated that links the identifiable information to the study code and this will be held separately and securely on a password protected PC also at the Westmead Applied Research Centre. Your information will only be used for the purpose of this research project and it will only be disclosed with your permission, except as required by law.

It is anticipated that the results of this research project will be published and/or presented in a variety of forums. In any publication and/or presentation, information will be provided in such a way that you cannot be identified.

In accordance with relevant Australian privacy and other relevant laws, you have the right to request access to your information collected and stored by the research team. You also have the right to request that any information with which you disagree be corrected. Please contact Dr Shalinie King if you would like to access your information.

Any information obtained for the purpose of this research project and for the future research described in Section 16 that can identify you will be treated as confidential and securely stored. It will be disclosed only with your permission, or as required by law.

**15 Complaints and compensation**

If you suffer any injuries or complications as a result of this research project, you should contact the study team as soon as possible and you will be assisted with arranging appropriate medical treatment. If you are eligible for Medicare, you can receive any medical treatment required to treat the injury or complication, free of charge, as a public patient in any Australian public hospital.

**16 Who is organising and funding the research?**

This research project is a collaborative research project between Professor Clara Chow from the Western Sydney Local Health District, Professor Axel Spahr, Dr Shalinie King, and Lauren Church from the University of Sydney.

**17 Who has reviewed the research project?**

All research in Australia involving humans is reviewed by an independent group of people called a Human Research Ethics Committee (HREC). The ethical aspects of this research project have been approved by the HREC of the Western Sydney Local Health District.

This project will be carried out according to the *National Statement on Ethical Conduct in Human Research (2007)*. This statement has been developed to protect the interests of people who agree to participate in human research studies.

**18 Further information and who to contact**

The person you may need to contact will depend on the nature of your query.

If you want any further information concerning this project or if you have any medical problems which may be related to your involvement in the project (for example, any side effects), you can contact the principal study doctor, Professor Clara Chow, or any of the following people:

**Clinical contact person**

| Name | Dr Shalinie King |
| --- | --- |
| Position | Investigator |
| Telephone | 02 9351 8344 |
| Email | Shalinie.king@sydney.edu.au |

For matters relating to research at the site at which you are participating, the details of the local site complaints person are:

**Complaints contact person *Westmead***

| Position | Patient Experience Unit |
| --- | --- |
| Telephone | 8890 7014 |
| Email | Wslhd-westmead-feedback@health.nsw.gov.au |

**Complaints Contact person** ***Blacktown***

| Position | Office of the General Manager |
| --- | --- |
| Telephone | 9881 8000 |
| Email | wslhd-bmdhexec@health.nsw.gov.au |

If you have any complaints about any aspect of the project, the way it is being conducted or any questions about being a research participant in general, then you may contact:

| Reviewing HREC name | *WSLHD HREC Committee* |
| --- | --- |
| HREC Executive Officer | *Kellie Hansen* |
| Telephone | *8890 9007* |
| Email | *Wslhd-researchoffice@health.nsw.gov.au* |

**Reviewing HREC approving this research** **and HREC Executive Officer details**

| WMD Research Office | WSLHD Research Governance Office |
| --- | --- |
| Telephone | 02 8890 9007 |
| Email | Wslhd-researchoffice@health.nsw.gov.au |

**HREC Office contact (Single Site -Research Governance Officer)**

**Consent Form -** *Adult providing own consent*

| **Title** | Oral hygiene instruction and digital oral health education within cardiac rehabilitation clinics, a novel approach to improving oral and cardiovascular health. |
| --- | --- |
| **Short Title** | Health impacts on the heart, the mouth is where it starts |
| **Principal Investigator** | Professor Clara Chow |
| **Location** | Westmead and Blacktown Cardiac Rehabilitation Facilities. |

**Declaration by Participant**

- I have read the Participant Information Sheet or someone has read it to me in a language that I understand.
- I understand the purposes, procedures and risks of the research described in the project.
- I give permission for my doctors, other health professionals, hospitals, or laboratories outside this hospital to release information to The University of Sydney concerning my disease and treatment for the purposes of this project. I understand that such information will remain confidential.
- I acknowledge that any regulatory authorities may have access to my medical records specifically related to this project to monitor the research in which I am agreeing to participate. However, I understand my identity will not be disclosed to anyone else or in publications or presentations.
- I have had an opportunity to ask questions and I am satisfied with the answers I have received.
- I freely agree to participate in this research project as described and understand that I am free to withdraw at any time during the study without affecting my future health care.
- I understand that I will be given a signed copy of this document to keep.

|  | | | | | | |
| --- | --- | --- | --- | --- | --- | --- |
|  | Name of Participant (please print) | |  |  |  |  |
|  | | | | | | |
|  | Signature |  | | Date |  |  |
|  | | | | | | |

**Declaration by Study Doctor/Senior Researcher^†^**

I have given a verbal explanation of the research project, its procedures and risks and I believe that the participant has understood that explanation.

|  | | | | | | |
| --- | --- | --- | --- | --- | --- | --- |
|  | Name of Study Doctor/  Senior Researcher^†^ (please print) | |  | | |  |
|  | | | | | |  |
|  | Signature |  | | Date |  |  |
|  | | | | | | |

^†^ A senior member of the research team must provide the explanation of, and information concerning, the research project.

Note: All parties signing the consent section must date their own signature.

I consent to the storage and use of my clinical examination and survey data taken from me for use, as described in the relevant section of the Participant Information Sheet, for:

• This specific research project

• Other research that is closely related to this research project

• Any future research.

|  | | | | | | |
| --- | --- | --- | --- | --- | --- | --- |
|  | Name of Participant (please print) | |  | | |  |
|  | | | | | | |
|  | Signature |  | | Date |  |  |
|  | | | | | | |

|  | | | | | | |
| --- | --- | --- | --- | --- | --- | --- |
|  | Name of Study Doctor/  Senior Researcher^†^ (please print) | |  | | |  |
|  | | | | | |  |
|  | Signature |  | | Date |  |  |
|  | | | | | | |

^†^ A senior member of the research team must provide the explanation of and information concerning the research project.

Note: All parties signing the consent section must date their own signature.

**Form for Withdrawal of Participation -** *Adult providing own consent*

| **Title** | Oral hygiene instruction and digital oral health education within cardiac rehabilitation clinics, a novel approach to improving oral and cardiovascular health. |
| --- | --- |
| **Short Title** | Health impacts on the heart, the mouth is where it starts |
| **Principal Investigator** | Professor Clara Chow |
| **Location** | Westmead and Blacktown Cardiac Rehabilitation Facilities. |

**Declaration by Participant**

I wish to withdraw from participation in the above research project and understand that such withdrawal will not affect my routine treatment, my relationship with those treating me or my relationship with Westmead or Blacktown Hospitals.

|  | | | | | | |
| --- | --- | --- | --- | --- | --- | --- |
|  | Name of Participant (please print) | |  |  |  |  |
|  | | | | | | |
|  | Signature |  | | Date |  |  |
|  | | | | | | |

*In the event that the participant’s decision to withdraw is communicated verbally, the Study Doctor/Senior Researcher will need to provide a description of the circumstances below.*

|  |
| --- |

**Declaration by Study Doctor/Senior Researcher^†^**

I have given a verbal explanation of the implications of withdrawal from the research project and I believe that the participant has understood that explanation.

|  | | | | | | |
| --- | --- | --- | --- | --- | --- | --- |
|  | Name of Study Doctor/  Senior Researcher^†^ (please print) | |  | | |  |
|  | | | | | |  |
|  | Signature |  | | Date |  |  |
|  | | | | | | |

^†^ A senior member of the research team must provide the explanation of and information concerning withdrawal from the research project.

Note: All parties signing the consent section must date their own signature.
