# Supplementary material for: Evaluating the impact of oral hygiene instruction and digital oral health education within cardiac rehabilitation clinics: A protocol for a novel, dual centre, parallel randomised controlled trial
Source: PLoS One. 2024 Jul 11;19(7):e0306882. doi: 10.1371/journal.pone.0306882 (PMC11239009; doi:10.1371/journal.pone.0306882)
Supplement: S4 File — (PDF) [file pone.0306882.s004.pdf]

Cardiac Nurse SurveyOral hygiene instruction and digital oral health education within cardiac rehabilitation clinics, a novel approach to improving oral and cardiovascular health.

Demographics

Age

____

Sex

◯ Male ◯ Female

Have you received oral care training?

◯ Yes ◯ No

If yes, where did you receive your training:

☐ TAFE
☐ Oral health practitioner
☐ Within the hospital system/on the ward
☐ University/part of degree
☐ Other ______

Confidence in patients' oral health

An oral health check should be completed during patients' initial CEAP assessment

◯ Yes ◯ No

If an oral check were completed in CEAP, which factors do you think should be evaluated during it? (select any that you feel is correct)

☐ Presence of natural teeth
☐ Teeth health status
☐ Gum health status
☐ Presence of oral mucosal lesions
☐ Presence, integrity and hygienic status of partial or complete denture(s)
☐ Presence of oral pain and/or burning
☐ All of the above

Oral Health Confidence

	Strongly Agree	Agree	Neutral	Disagree	Strongly Disagree
I am confident in identifying oral health problems in cardiac patients.	◯	◯	◯	◯	◯
I am confident I can give the right oral health advice to cardiac patients:	◯	◯	◯	◯	◯

I am confident to make a referral to an oral health practitioner for my patients	◯	◯	◯	◯	◯

I have access to oral health promotional resources

◯ Yes ◯ No

Do you ever have oral health complaints from your patients?

◯ Yes ◯ No

If yes, what are they:

- ☐ Bleeding gums
- ☐ Toothache
- ☐ Cavities
- ☐ Loose teeth
- ☐ Sensitivity (E.g. pain with hot or cold)
- ☐ Teeth that don't look right
- ☐ Dry mouth
- ☐ Other ______

How important is a healthy mouth for overall health?

◯ Not important ◯ Somewhat important ◯ Neutral ◯ Important ◯ Very important

Oral Health on Cardiovascular Health

	Yes	No	Unsure
Brushing teeth and gums should be completed 2 x daily using fluoridated toothpaste.	◯	◯	◯
Flossing should be done daily to clean in between teeth	◯	◯	◯
People with heart problems should avoid dental treatment	◯	◯	◯
People with existing heart problems should visit a dentist regularly for a check up	◯	◯	◯
People with heart problems should only see a dentist when there is an emergency	◯	◯	◯
Poor oral health may affect an existing heart condition	◯	◯	◯
Some medications for high blood pressure can cause people to experience dry mouth	◯	◯	◯
Dry mouth does not increase the risk of dental decay	◯	◯	◯

Some medications for high blood pressure can affect the sense of taste	◯	◯	◯
Some heart medications can cause swelling or overgrowth (thickening) of the gums	◯	◯	◯
The overgrowth of gums can lead to poor oral health	◯	◯	◯
Bad breath is a sign of gum disease	◯	◯	◯
Loose teeth is one sign of severe gum disease	◯	◯	◯

Access to Oral Health Practitioner in CEAP

Should nurses be providing oral health education, assessment and referrals to CEAP patients?

◯ Yes. ◯ No. ◯ Unsure

Why:

____

Do you think it would be beneficial for an oral health practitioner to assess patients' oral health in CEAP?

◯ Yes. ◯ No. ◯ Unsure

Why

____

Perception of OHE CVD program in CEAP

	Strongly Agree	Agree	Neutral	Disagree	Strongly Disagree
This program has raised oral health awareness in the patient population	◯	◯	◯	◯	◯
This program has raised oral health awareness within the staff	◯	◯	◯	◯	◯
Digital oral health education is beneficial to patients	◯	◯	◯	◯	◯

Do you have any feedback or suggestions on how this form of oral health education could be improved?

____
