# Supplementary material for: Evaluating the impact of oral hygiene instruction and digital oral health education within cardiac rehabilitation clinics: A protocol for a novel, dual centre, parallel randomised controlled trial
Source: PLoS One. 2024 Jul 11;19(7):e0306882. doi: 10.1371/journal.pone.0306882 (PMC11239009; doi:10.1371/journal.pone.0306882)
Supplement: S5 File — (PDF) [file pone.0306882.s005.pdf]

	STUDY PERIOD					
	Enrolment	Allocation	Post-allocation			Close-out
**TIMEPOINT** **Weeks**	***Pre-randomisation***	**Before intervention**	***Baseline***	***6 weeks***	***12 weeks***	***12-week follow up***
**ENROLMENT:**						
**Eligibility screen**	X					
**Informed consent**	X					
Survey, SBI, API, PSR	X					
**Randomisation**		X				
**Allocation**		X				
**INTERVENTIONS:**						
Group A OHI DOHE			X	X	X	X
Group B DOHE only			X	X	X	X
Group C Control			X	X	X	X
**ASSESSMENTS:**						
PSR			X			
API/SBI			X	X	X	X
Survey: OH perceptions & knowledge			X	X	X	X
Survey: OH habits			X	X	X	X
Survey: OH motivations & confidence			X	X	X	X
Survey: PT perceptions in of nurses in OH			X			
Survey: OH practitioner attendance			X			
Survey: PT motivation to see OH practitioner			X	X	X	X

* API: approximal plaque index, DOHE: digital oral health education, OH: oral health, OHI: oral hygiene instruction, PSR: periodontal screening and recording, PT: patient. SBI: sulcus bleeding index.
