# Supplementary material for: Evaluating the impact of oral hygiene instruction and digital oral health education within cardiac rehabilitation clinics: A protocol for a novel, dual centre, parallel randomised controlled trial
Source: PLoS One. 2024 Jul 11;19(7):e0306882. doi: 10.1371/journal.pone.0306882 (PMC11239009; doi:10.1371/journal.pone.0306882)
Supplement: S1 Protocol — (DOCX) [file pone.0306882.s006.docx]

**Title:**

Oral hygiene instruction and digital oral health education within cardiac rehabilitation clinics, a novel approach to improving oral and cardiovascular health.

**Short Title:**

Health impacts on the heart, the mouth is where it starts.

**Principal Investigator:** Professor Clara Chow^1,2^

**Associate Investigators:** Professor Axel Spahr^1^, Professor Janet Wallace^1^, Dr Shalinie King^1,2^, Lauren Church PhD Candidate^1,2^.

**Affiliations:**

1 - The University of Sydney Dental School

2 - Westmead Applied Research Centre

**Introduction**

**Periodontal Disease**

All dentated individuals are susceptible to periodontal disease (1). The most common form, gingivitis, is estimated to affect up to 90% of any population (2); whilst periodontitis, more destructive in nature, affects close to 50% of the global population (2). Periodontal disease is defined as a multifactorial inflammatory process involving dysbiotic biofilm (3), a well organised colony of bacteria(1); that, if left untreated, can lead to destruction of the supporting structures of the teeth including: connective tissue, periodontal ligament fibres, and alveolar bone (4,5). The pathogenesis of this destruction is characterised by the combination of dysbiotic biofilm; commonly poor oral hygiene, time, allowing the oral biofilm or it’s bacteria to invade deeper into gingival tissues, and a hyperactive immune host response (1).

Gingivitis, the first stage of periodontal disease, initiates within 24-48hrs after ceasing toothbrushing (6). As the biofilm and its secretions are allowed to remain in contact with gingival tissues, even at this early stage the immune response is activated (7). If these bacterial deposits are allow to remain insitu, the predominately supragingival, aerobic gram-positive biofilm will slowly migrate deeper into the gingival sulcus and will, within 3 weeks, transform into a mostly anaerobic gram-negative biofilm(1,8). At this stage in the disease process, the immune response to the bacteria and their exo and endotoxins is at its most powerful releasing proinflammatory cytokines IL-1α , IL-1β, TNF-α, IL-8, IL-1ra, IL-10, and IL-12, matrix metalloproteinases (MMPs) and prostaglandin E2 (PGE_2_) as well as T and B cell lymphocytes, polymorphonuclear leukocytes (PMNs). For most individuals, the battle of bacteria and immune response remains even. However, in susceptible individuals their immune response intensifies in a way that the level of destruction outweighs repair (1,6,8).

This irreversible damage can have severe implications on quality of life (QoL) as it can involve tooth mobility and eventual loss; affecting one’s speech, eating, and causing psychological damage from fear of the judgement of others (9,10). Once destruction is evident, the condition is classified as periodontitis, the sixth most prevalent chronic condition worldwide (11). In Australia, the incidence of periodontitis is predicted to increase, with current figures showing just over 30% of the population is affected (12). Whilst the latest data shows between 1990-2019 severe periodontitis incidence has increased by 8.44% globally (13).

**Inflammation and Cardiovascular Disease**

Once triggered by periodontal disease, the immune response has systemic implications evidenced by an increase in specific inflammatory markers such as high-sensitive C-reactive protein (hsCRP) and IL-6 (14) detected in blood samples. Following the termination of tooth brushing, one study demonstrated evidence of these markers after only 21 days in healthy, young participants (mean age 23.35 ± 3.64 years) (7). The elevation of inflammatory markers, in particular hsCRP, is strongly associated with an increased risk of atherosclerosis (15), the primary cause of cardiovascular disease (CVD) (16).

Atherosclerosis is developed from matured atherosclerotic plaque forming on blood vessel walls (17). This fibro-lipid structure consists of key immune inflammatory cells and markers such as leukocytes, cytokines, chemoattractants, and MMPs (18). Inflammation as such has been identified as being heavily involved in all stages of atherosclerosis formation (19). Oral biofilm is also recognised as an influencing factor (18,20) and has been speculated to initiate or promote atherosclerotic plaque formation. Resulting from the bacterial challenge in the oral cavity, vasodilation in gingival tissues (1) allows the virulent bacteria, their toxins as well as the induced proinflammatory mediators easy access to body organs and systems via the bloodstream (4,5,21,22). Once transported, they can influence or cause harm to susceptible tissues and introduce localised inflammatory responses elsewhere in the body (23) and as such, as well as CVD (21) are linked to stroke (20), Alzheimer’s disease, diabetes, and cancer (5).

Patients living with CVD are at a high risk of having a cardiac event with even the slightest elevation of inflammatory markers (24,25) and have an increased risk of recurrent cardiovascular events, including death after surviving a myocardial infarction (MI) (26). In 2021, CVD (ICD20-ICD25) was the leading cause of death in Australia (27) and is responsible for 32% of deaths and 38% of premature deaths globally (28). Due to the increased systemic inflammation caused by periodontal disease (29), CVD patients who are also suffering with periodontal disease, have a further elevated risk of a future cardiac event. Therefore, it is integral to reduce the inflammatory comorbidity of periodontal disease to help prevent poor CVD outcomes.

**Prevention**

Primary prevention of periodontal disease

The most important and effective way to prevent periodontal disease is with an optimal oral hygiene routine (30), followed by regular dental check-ups and hygiene appointments, as well as the absence or control of modifying risk factors such as smoking (31), and diabetes (32). Poor oral hygiene habits, defined as never or rarely brushing teeth, have been shown to increase the risk of a CVD event significantly (HR 1.7, 95% CI 1.3 to 2.3; P<0.001) (33), whilst incorporating just one toothbrushing session per day is associated with a 9% risk reduction of a CVD event (34). The recommended ideal oral hygiene routine includes brushing twice a day for at least two minutes with a fluoridated toothpaste (35,36), preferably with an electric oscillating/rotating power toothbrush (37–39). As adjunct, as toothbrushes cannot reach all tooth surfaces, particularly in between the teeth, interdental cleaning products such as interdental brushes should be used before brushing at least once daily (40–42).

Essential to good oral hygiene and the prevention of oral disease, is education (43). Involving CVD patients after having a MI, a randomised controlled trial (RCT) attempted to introduce an intervention directly to patients in a cardiology ward (44) as a way of lowering their future CVD event risk. However, due to a lack of awareness of the importance of oral health for cardiovascular health, recruitment has been low. It is reasonable that there is a lack of understanding of the importance of oral health for cardiovascular health. Currently, the most easily accessible health information regarding ways to reduce risk factors attaining to CVD only includes smoking cessation, reducing alcohol intake, improving diet, and becoming physically active (28,45–49). Unless specifically searched for, the importance of good oral hygiene for cardiovascular health is never mentioned.

**Oral health promotion studies**

Australians have a universal right to all areas of healthcare (50), and should have access to any health services needed, whenever and wherever they are required (51). However, oral health is not a priority within hospital wards currently, and there is no plan to incorporate an oral health practitioner (OHP) into routine care in hospitals as part of Australia’s National Oral Health Plan 2015–2024 (52). Oral care is a part of standard care within hospitals, and when appropriately completed, it has shown to reduce secondary illness incidence (53). Traditionally, oral health care and education is provided by an OHP. However, in hospitals this is designated to nurses who have many barriers to overcome to deliver oral health education, such as: a lack of training, time or resources, to name just a few (54–56). Additional obstacles can present from patients, especially if oral health is not a priority to them; but can also derive and can come from the hospital organisations themselves (55). OHPs as such, are an appropriate alternative to take on this responsibility and could help to eliminate barriers, alleviate the workload of nurses, and improve oral health knowledge by delivering education directly to patients, providing access to resources, and, if required, refer for any urgent oral care (57).

Many interventional studies regarding oral care interventions have been conducted residential aged care facilities (RACF) (58–61). One 6-month study placed an OHP within five RACFs on the Central Coast, NSW, Australia, and trialed a program that included individualised oral health risk assessments, health care plans, oral hygiene instructions, and referral for extensive dental treatment when necessary (62). Additionally, an informal oral health training was provided to all RACF staff members. The positive health outcomes demonstrated by this 6-month interventional study, known as ‘Senior Smiles’, have resulted in the immediate incorporation of this concept in two RACFs and the number of further RACFs adopting this concept is continuously growing (63).

Conversely, at present, only minimal oral health interventions have been conducted within hospital wards, clinics, or outpatient facilities. Those that have been conducted mainly involve the education of nurses (53,64–66), but are not empowering patients. The limited literature available regarding oral health interventions direct to patients include: a cardiology ward pre- and post-heart surgery (67), a stroke ward (68), and a diabetic outpatient clinic (69). Interestingly most oral care intervention studies have been conducted within mental health wards (70–73). Interventions within a cardiac rehabilitation out-patient facility exist, however they are only related to self-reported oral health knowledge and behaviour (74,75). Currently, there are no known interventions clinically monitoring the oral hygiene status of CVD patients in a cardiac rehabilitation out-patient setting.

The use of digital media has been proven effective as a strategy to improve patient’s knowledge and confidence regarding their health condition (76,77), even in individuals with low literacy levels (78,79). Additionally, digital media based education strategies in the dental clinic waiting room have shown to improve self-reported oral hygiene habits (80). However, to the best of our knowledge, there are no published studies seeking to improve oral health knowledge, motivation, and confidence by using a digital education approach in a cardiac rehabilitation setting. The aim of this study is to assess whether incorporating an individualised oral hygiene instruction (OHI) partnered with a digital oral health education (DOHE) package can improve oral hygiene and clinic oral health of CVD patients in cardiac rehabilitation out-patient facilities.

The primary outcome of this study is to determine if individualised OHI combined with DOHE improves approximal plaque index (API) (81) scores between baseline and the 6-week follow-up, compared with usual care/no OHI education. Secondary outcomes of this study will determine if digitally delivered oral health education alone or partnered with individualised OHI improves API scores between baseline and the 12-week follow-up, compared with usual care. It will compare the mean reduction of API and SBI between each group at baseline and follow up, and determine if any participant reaches scores that are clinically significant. It will assess any changes to patients’: motivation, confidence, and behaviour regarding their oral hygiene practices, knowledge of oral health impacts on cardiovascular health, as well as their experience with oral care whilst on the ward. Additionally, it will assess the cardiac nursing staff views on an oral health education program within the rehabilitation facility. As good oral hygiene habits are an upstream approach of reducing risk of systemic chronic inflammation, and as such, risk of a future cardiac event (29), we hypothesise introducing an individual OHI partnered with DOHE program into the cardiac rehabilitation out-patient clinic will help improve API scores between baseline and follow-up. We also hypothesise the program will increase patients’ awareness of the impact of oral health on cardiovascular health and help to motivate and give them confidence in caring for their oral health; and if not doing so already, incorporating regular visits to an OHP.

**Methods**

Study Design

A dual-center, single-blind (examiner), parallel design randomised control trial of 165 patients attending an outpatient cardiac rehabilitation center within two public hospitals in Sydney, Australia. See **Figure 1.** for study flow diagram. The intervention populations will receive either a tablet-delivered specifically selected series of advertisement free, web-based educational videos covering multiple CVD and oral health related topics alone or partnered with individually tailored OHI. The control population will receive usual care as detailed below. The examiner will be blinded to the group allocation.

Patient population

The study population will be patients presenting to the outpatient cardiology rehabilitation centre participating in the Cardiac Education and Assessment Program (CEAP) at Westmead and Blacktown public hospitals, Sydney Australia. CEAP provides personalised support, exercise and education to help strengthen patient’s hearts after having a cardiovascular event and helps to lower risk of a future cardiovascular event (82). These patients attend due to a range of heart conditions such as coronary heart disease, heart failure, and those waiting for, or who have had a heart transplant. Due to the variety of heart conditions and co-morbidities, patients are placed on either a 6-week or 12-week program and can attend once or twice weekly. As these patients have taken part in this program, prioritising their cardiovascular health, and showing a motivation to reduce future risk of a cardiovascular event, we predict these patients will be interested in receiving education regarding oral health impacts on CVD.

Participant eligibility

A total of 165 participants will be recruited at either Westmead or Blacktown outpatient cardiac rehabilitation clinics who are attending as part of the CEAP program. Patients 18 years or older with a recent diagnosis of CVD , recent myocardial infarction and/or recent hospitalisation resulting from CVD will be approached for screening. This will include a clinical examination consisting of periodontal screening and recording (PSR), API, and sulcus bleeding index (SBI) (83) will be completed, followed by a self-report questionnaire. Patients will be excluded if:

- they have been diagnosed with heart failure.
- have/are waiting for a heart transplant,
- are too physically or mentally unwell on the day.
- have a cognitive impairment.
- who do not speak or have limited language skills in English.

If they have visited an OHP for treatment of gum disease (supra- and/or subgingival scaling and root planning) in the last 6 months,

- have an API score of ≤59%
- are edentulous.

A study researcher will ensure that eligibility criteria are satisfied before approaching the participants by viewing their clinical records. Part of discussing the study with participants will allow the researcher to determine if they are edentulous or have seen an OHP within the last 6 months which will exclude them before the baseline assessment. A sequence of the recruitment process is provided in **Fig 2**.

Recruitment and consent

Eligible patients will be recruited either before or after their CEAP session in the cardiac rehab clinic. A trained oral health therapist will explain the study to potential participants. Electronic consent forms will be provided on iPads provided by the study, being hosted on Western Sydney Local Health District (WSLHD) Research Electronic Data Capture (REDCap) (84). As such the participant must complete written/electronic consent before being able to progress to the clinical examination (API, SBI and PSR) and data collection stage. Ethical approval will be obtained via: Western Sydney Local Health District Human Research Ethics Committee (HREC). This trial will be registered on the Australian New Zealand Clinical Trails Registry.

Randomisation

Randomisation will be stratified by age and site. A randomisation allocation table will be computer generated and REDCap will be used for allocation of participants to each group. Participants will be randomised at the end of the self-report questionnaire and randomised into a 1:1:1 ratio into one of three arms of the study. Due to the nature of the interventions within the trial, participants cannot be blinded.

Interventions

Participants will be randomised into one of 3 arms as follows: Group A) individualised OHI combined with an oral health video package, Group B) oral health video package only, and Group C) usual care without any oral health education given. At the final follow up, after completing clinical measurements and the follow-up questionnaire, Group B and Group C will be given tailored OHI and Group C will also be shown the video package to ensure all participants have received the comprehensive oral health educational package after completion of the study. A single calibrated and trained oral health therapist, blinded to the treatment allocations will perform the baseline and follow up assessments on all participants. After the initial assessment, if any obvious dental issues (without the use of radiographs) are detected, patients will be given a letter that can be taken to a dental practitioner for further investigation.

Oral hygiene instruction

Trained research assistants, oral health therapy and/or dental students will deliver tailored OHI, using API and SBI results from the assessment as a guide for patient focus. Toothbrushing or interdental cleaning instructions will be given in accordance with the oral hygiene standard operating procedure (SOP). See separate document.

Video packages

Videos to be included in the oral health education package will be evaluated and rated by a multi-disciplinary panel to ensure content validity and consumer engagement. The intervention video package will include short clips in relation to oral health and CVD, as well as oral hygiene practices. (77). The videos included will be advertisement free; and created by either government bodies, or relevant health bodies such as the Australian Dental Association (ADA). These will be delivered to participants in Group A and Group B once the self-report form has been completed at baseline, and Group C at the final visit, embedded within REDCap.

**Study outcomes**

Primary outcome

The primary outcome of this study is to show the percentage of participants reducing their API score ≥40% after 6 weeks, compared to baseline is higher in Group A than in Group C.

Secondary outcomes

The secondary outcomes of the study will be:

- To show the percentage of participants reducing their API score by ≥40% after 12 weeks, compared to baseline, is higher in Group A (OHI & DOHE) than in Group C (usual care/no oral hygiene intervention).
- To show the reduction in mean API score after 6 weeks, compared to baseline, is higher in Group A (OHI & DOHE) than in Group C (usual care/no oral hygiene intervention).
- To show the reduction in mean API score after 12 weeks, compared to baseline, is higher in Group A (OHI & DOHE) than in Group C (usual care/no oral hygiene intervention).
- To show the reduction in mean API score after 6 weeks, compared to baseline, is higher in Group A (OHI & DOHE) than in Group B (DOHE).
- To show the reduction in mean API score after 12 weeks, compared to baseline, is higher in Group A (OHI & DOHE) than in Group B (DOHE).
- To show the reduction in mean API score after 6 weeks, compared to baseline, is higher in Group B (DOHE) than in Group C (usual care/no oral hygiene intervention).
- To show the reduction in mean API score after 12 weeks, compared to baseline, is higher in Group B (DOHE) than in Group C (usual care/no oral hygiene intervention).
- To show the reduction in mean SBI score after 6 weeks, compared to baseline, is higher in Group A (OHI & DOHE) than in Group C (usual care/no oral hygiene intervention).
- To show the reduction in mean SBI score after 12 weeks, compared to baseline, is higher in Group A (OHI & DOHE) than in Group C (usual care/no oral hygiene intervention).
- To show the reduction in mean SBI score after 6 weeks, compared to baseline, is higher in Group A (OHI & DOHE) than in Group B (DOHE).
- To show the reduction in mean SBI score after 12 weeks, compared to baseline, is higher in Group A (OHI & DOHE) than in Group B (DOHE).
- To show the reduction in mean SBI score after 6 weeks, compared to baseline, is higher in Group B (DOHE) than in Group C (usual care/no oral hygiene intervention).
- To show the reduction in mean SBI score after 12 weeks, compared to baseline, is higher in Group B (DOHE) than in Group C (usual care/no oral hygiene intervention).
- Assess the percentage in each Group that has reached clinically significant API scores of ≤35%.
- Assess the percentage in each Group that has reached clinically significant SBI scores of ≤25%.
- Assess the percentage in each Group that has reached clinically significant API scores of ≤35% as well as a clinically significant SBI scores of ≤25%.
- Improved oral hygiene habits as reported by an increase in brushing frequency and/or inclusion of interproximal cleaning, comparing baseline to follow-up.
- Improved understanding between the link between heart health and oral health (Likert scale). A yes response increase of ≥ 5 out of 12 will be considered as an improved understanding.
- Confidence to improve oral hygiene habits with a self-reported 5-point Likert scale. A change in response from neutral, disagree or strongly disagree to agree or strongly agree compared to baseline will be considered confident.
- Motivation to improve their oral health and see an OHP regularly with a yes/no response and a self-reported 5-point Likert scale. Answer change from no to yes will be considered motivated to improve their oral health and a response of: ‘Yes I have made an appointment since the assessment’ or ‘Yes, however I have not yet made an appointment’ will be considered motivated to visit an OHP regularly.
- Patient perceptions of a nurse’s role in their oral health care using yes, no answers. Answering yes will be considered cardiac nurses can play a role in their oral health.
- Cardiac nurse perception of the importance and effectiveness of the intervention in the rehab setting (Likert scale). A response of 4 or 5 will be considered as the intervention being important and effective.

**Data collection and management**

Data will be collected at baseline, 6 weeks and at the final 12-week assessment and stored via REDCap. Baseline data will include demographic information, medical history, weight (kg), height (cm), body-mass index (BMI), oral hygiene practices, and oral hygiene knowledge associated with CVD. CEAP nurses record weight, height, and BMI when patients first attend the program, as such this data and other required medical data will be retrieved from participant clinical records. Questionnaires delivered via REDCap via iPads. Clinical parameters will be recorded at baseline, the 6-week, and the final follow-up after 12 weeks. PSR, SBI, and API will be completed using a disposable World Health Organisation (WHO) periodontal probe and mirror, with tricolour disclosing gel. For timeline and mode of collection see **Table 1**.

Each site will hold a master spreadsheet held on WSLHD servers. Identifiable data including name, address and phone numbers will be housed on secure servers within the WSLHD network. The server is managed and maintained by WSLHD Digital Health Services (DHS). Each patient will be assigned a code – the patient’s name and dental record number will be linked to the study code and stored in an encrypted and password protected folder, separate to all other data files, with access to this folder limited to the principal investigator and coordinating principal investigator. All other data files will contain only the study code. Participant consent forms will be in digital form as part of REDCap, thus stored electronically. Data sheets will contain the study code, age and sex and will be stored electronically. De-identified data will be used for any analyses and publications and all data collected during the study will be retained for a period of 5 years following the study completion. We anticipate no harm resulting from the intervention, as such we do not require a data safety monitoring board.

**Table 1.** Timing and mode of collection of study data

| Measurements and Surveys | Baseline | 6-week  Assessment | 12-week  Final Assessment |
| --- | --- | --- | --- |
| Demographic information and medical history and behaviour/risk factors | X |  |  |
| Clinical measurements PSR/API/SBI | X | X | X |
| Oral health Perceptions and knowledge | X | X | X |
| Oral hygiene habits | X | X | X |
| Motivation and confidence and to improve oral hygiene practices | X | X | X |
| Perceptions of nurses involvement oral health care | X |  |  |
| Do they have an oral health practitioner and see them regularly | X |  |  |
| Perceived value of their oral health | X | X | X |
| Motivation to see oral health practitioner | X | X | X |
| *Perception of cardiac nurses of importance and effectiveness of the intervention – delivered to nurses at study completion. | | | |

**Clinical measurements**

API and SBI are a rapid way of assessing and monitoring a patient’s oral hygiene (81,83). Using the WHO periodontal probe again, SBI is completed by gently inserting the probe into the interproximal areas of the buccal sulcus of the first and fourth quadrant, and palatal/lingual sulcus surfaces of the second and third quadrant. Any evidence of bleeding on probing (BOP) is recorded. Next, API is completed. Using a micro brush, place a small amount of the disclosing liquid on and around the interdental papilla of each tooth. This occurs on the buccal surfaces of the second and third quadrants, and the lingual/palatal surfaces of the first and fourth quadrants. After allowing the patient to rinse, interproximal sites that have any remaining disclosing solution is recorded as a positive reading.

Assessing oral hygiene using SBI and API requires a percentage to be calculated. See **Table 2** for API/SBI percentage results. For the SBI percentage, divide the number of positive BOP sites by the number of total tooth sites and times by 100. To work out API percentage divide the number of positive disclosing sites by number of tooth total sites, and times by 100. This will be completed at baseline and at the 6 and 12-week follow ups where percentages will be compared for any differences. Once an oral hygiene routine has been implemented, improvements can be confirmed as early as 2 weeks (85).

PSR is also a rapid assessment of a patient’s periodontal health (86). It is completed systematically by dividing the mouth into sextants and, starting in sextant one, gently inserting a WHO periodontal probe into the gingival sulcus taking a 6-point measurement of each tooth. Specific markings on the probe relates to a measurement, indicating to the OHP which code to classify the sextant as, and if a full periodontal screening and analysis should be performed. See **Table 3** for code definitions. Any participants returning a PSR code of 3 or 4 will still be included in the study, however, will be strongly advised to seek an OHP for further assessment.

**Sample size**

The required sample size for this study is calculated to be a total of 165 (1:1:1 Group A: Group B: Group C). This calculation includes provision for a 10% attrition rate, two tailed tests, type 1 error of 5%, and will have 80% power to detect any significant difference between Group A and Group C. This calculation is based from an API score reduction of 25% in the intervention arm of Ziebolz, et al 2009 study (87). However, due to our exclusion criteria of ≤59% API score, we will expect to see a 40% decrease in Group A, compared to 15% in Group C. This current study is powered to detect a difference in percentage proportion of those between Group A and Group C however, has poor power to detect a difference between Group B and Group C.

| Table 2. Adapted API/SBI measurement results (88) | | |
| --- | --- | --- |
|  | **API** | **SBI** |
| Poor oral hygiene | >35% | >25% |
| Fair oral hygiene | 35% | 15-25% |
| Good oral hygiene | <25% | <15% |

| Table 3. Adapted PSR code definitions (86,89). | |
| --- | --- |
| Code | Clinical Signs |
| 0 | Absence of clinical signs: no calculus, or BOP  Coloured band on WHO probe completely visible. |
| 1 | No calculus.  BOP  Coloured band on WHO probe completely visible. |
| 2 | Supra and/or subgingival calculus  BOP  Coloured band on WHO probe completely visible. |
| 3 | Periodontal pocket 3.5mm-5.5mm  Coloured band on WHO probe partially visible |
| 4 | Periodontal pocket >5.5mm deep  Coloured band on WHO probe no longer visible |
| X | Sextant absent of dentition |
| * | Periodontal abnormalities which include:  Furcation involvement  Mobility  Mucogingival problems for example: exudate, severe oedema  >3.5mm recession  The * is recorded next to the sextant number code. E.g. “3*” |

**Statistical analysis**

The statistical plan for this study will be determined prior to study completion. Analysis of the difference in API will be according to the intention-to-treat principle where participants are analysed in the arm they have been allocated. The level of statistical significance will be set at p-value <0.05. The primary analysis will be an adjusted analysis of variance performed to assess differences between groups for API scores between baseline and follow-up. Covariates used for adjusted analyses will include age, sex, highest PSR code, level of education, smoking status, and number of teeth. Subgroup analysis of the intervention effect and age, sex, education, ethnicity, and cardiovascular disease presentation (coronary heart disease, heart failure, heart transplant list, heart transplant receiver) will also be explored. Outcome measures available at baseline (PSR, SBI and API) will also be included in the statistical model. An unadjusted analysis will be performed to assess differences between groups using a X^2^ test for binary (categorical) outcomes and independent sample t-tests for continuous outcomes.

**Process evaluation**

We aim to assess the effectiveness the intervention has or does not have on oral hygiene habits and knowledge for patients with CVD. A recruitment log will be kept, recording patients who do not wish to participate, or for those who are ineligible to. Part of the follow-up questionnaire will include a self-assessment of acceptability and the educational value of the study, including assessment of the included videos and it will attempt to identify any areas for improvement. Additionally, a member of the study team will contact participants via telephone at the end of the study to obtain their perception of the study intervention. An anonymous questionnaire, with implied consent by clicking on the link will also be given to the nursing staff to assess their oral knowledge, perception of the intervention’s importance and effectiveness, and ways it may be better incorporated into CEAP. As no relevant questionnaire exists to determine the outcomes of this study an adapted questionnaire was developed from previously established tools (90–93). This will an anonymous survey with implied consent by clicking on the survey link sent to all CEAP nursing staff at the conclusion of the study.

**Conclusion**

The link between oral health and CVD has been proven (18,21,29). For the average patient with CVD, the accessibility of information regarding the importance of this link and the impacts oral hygiene practices has on their cardiovascular health, are almost non-existent (28,45–49). As such, oral health knowledge and the importance of oral hygiene amongst this population is minimal (74). Digital educational programs have proved effective in increasing knowledge, confidence, and motivation to improve health outcomes (77), even when literacy levels are low (78,79). Therefore, this study will assess if the introduction of individualised OHI and a DOHE instrument will influence the API score, oral health, practices, knowledge, and motivation of CVD patients attending a cardiac rehab out-patient facility.

**Ethics**

Ethics approval will be sought from the WSLHD Human Research Ethics Committee. During recruitment, baseline and follow-up assessments, participants will be informed of their right to decline to take part or withdraw from the trial at any time. It will be highlighted that non-participation or withdrawal will in no way affect their current or future care at the hospital. The authors will seek approval to any variations required to be made to the protocol. Informed consent will be gained from all participants before being included in the study.

**Dissemination**

The results from this study will be published in peer reviewed journals and presented nationally and internationally at both oral health and cardiology conferences.

**
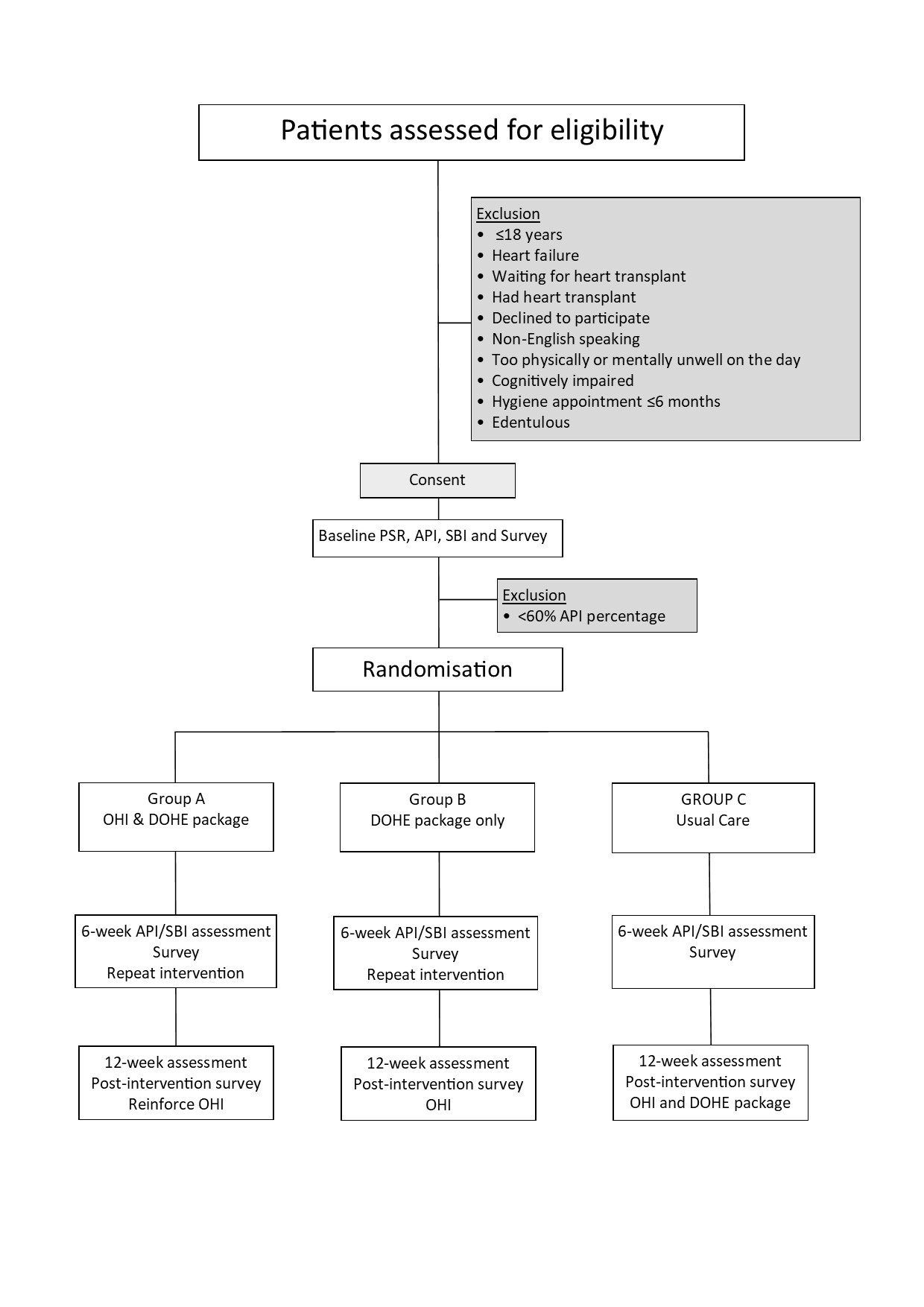
**

**Figure 1.** Study flow chart

**
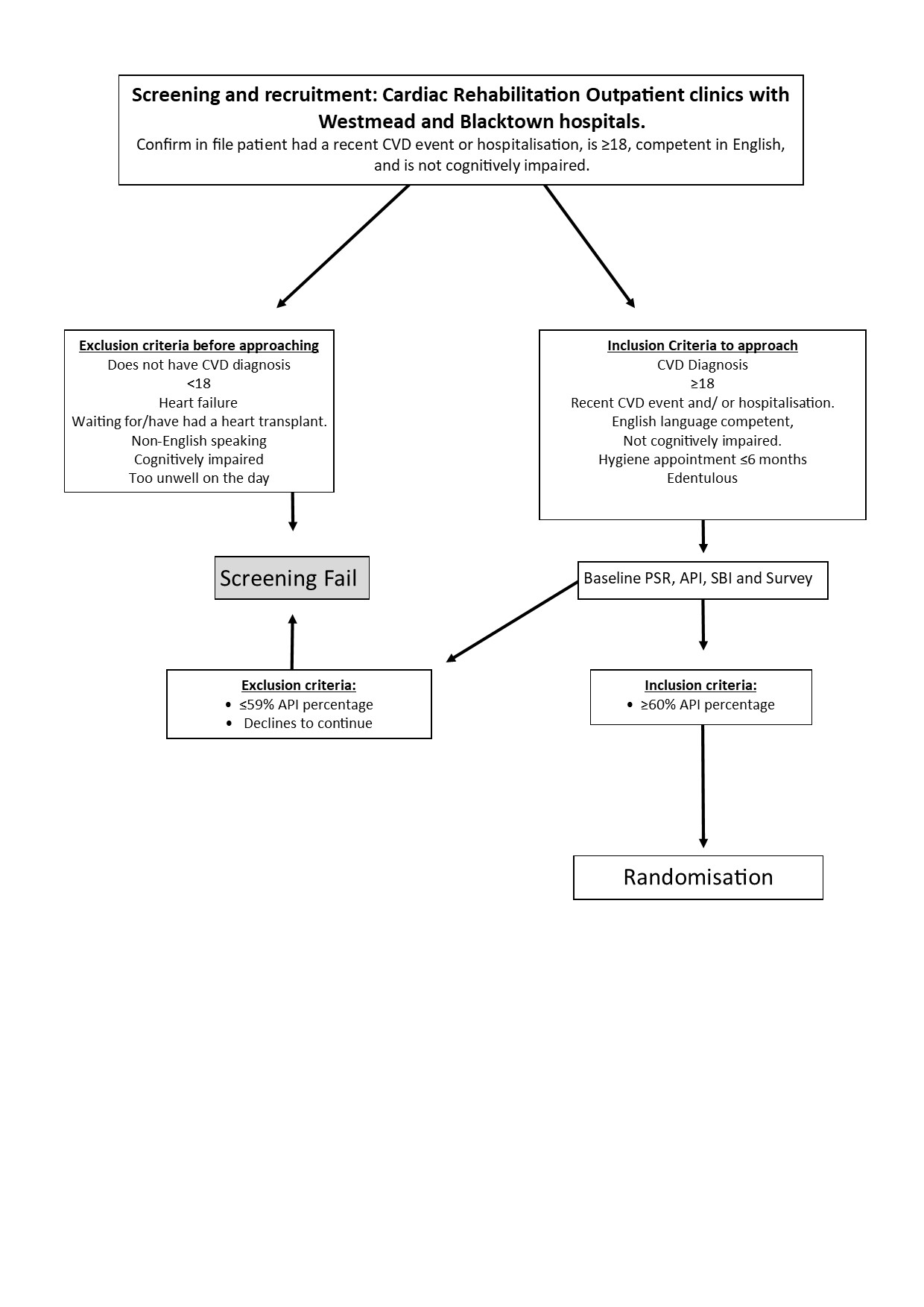
**

**Figure 2.** Patient recruitment flow diagram.

**References**

1. Nield-Gehrig J, Willman D. Host Immune Response to Periodontal Pathogens. In: Foundations of Periodontics for the Dental Hygienist. 3rd ed. Philadelphia: Wolters Kluwer | Lippincott WIlliams & Wilkins; 2011. p. 157–70.

2. Eke PI, Borgnakke WS, Genco RJ. Recent epidemiologic trends in periodontitis in the USA. Periodontol 2000. 2020 Feb 1;82(1):257–67.

3. Papapanou PN, Sanz M, Buduneli N, Dietrich T, Feres M, Fine DH, et al. Periodontitis: Consensus report of workgroup 2 of the 2017 World Workshop on the Classification of Periodontal and Peri-Implant Diseases and Conditions. J Periodontol. 2018;89(S1):S173–82.

4. Tonetti MS, Greenwell H, Kornman KS. Staging and grading of periodontitis: Framework and proposal of a new classification and case definition. J Clin Periodontol. 2018 Jun 1;45(S20):S149–61.

5. Bui FQ, Almeida-da-Silva CLC, Huynh B, Trinh A, Liu J, Woodward J, et al. Association between periodontal pathogens and systemic disease. Biomed J. 2019 Feb 1;42(1):27–35.

6. Alwaeli A. Anaerobic Bacteria Associated with Periodontitis. In: Bhardwaj S, editor. Oral Microbiology in Periodontitis [Internet]. 1st ed. IntechOpen; 2018 [cited 2022 Jul 15]. p. 19–32. Available from: https://www.intechopen.com/chapters/undefined/state.item.id

7. Eberhard J, Grote K, Luchtefeld M, Heuer W, Schuett H, Divchev D, et al. Experimental Gingivitis Induces Systemic Inflammatory Markers in Young Healthy Individuals: A Single-Subject Interventional Study. PLOS ONE. 2013 Feb 7;8(2):e55265.

8. Hasan A, Palmer RM. A clinical guide to periodontology: Pathology of periodontal disease. Br Dent J. 2014 Apr 25;216(8):457–61.

9. Wong LB, Yap AU, Allen PF. Periodontal disease and quality of life: Umbrella review of systematic reviews. J Periodontal Res. 2021 Jan 1;56(1):1–17.

10. Petersen P, Kwan S. The 7th WHO Global Conference on Health Promotion - towards integration of oral health (Nairobi, Kenya 2009). Community Dent Health. 2010 Jun 1;(27):129–36.

11. Kassebaum NJ, Bernabé E, Dahiya M, Bhandari B, Murray CJL, Marcenes W. Global Burden of Severe Periodontitis in 1990-2010: A Systematic Review and Meta-regression. J Dent Res. 2014 Nov 1;93(11):1045–53.

12. Do L, Luzzi L. Oral Health Status. In: Australia’s Oral Health: National Study of Adult Oral Health 2017-18 [Internet]. Adelaide: The University of Adelaide, South Australia; 2019. p. 38–96. Available from: https://www.adelaide.edu.au/arcpoh/national-study/report/Australias_Oral_Health_2017-18.pdf

13. Chen MX, Zhong YJ, Dong QQ, Wong HM, Wen YF. Global, regional, and national burden of severe periodontitis, 1990–2019: An analysis of the Global Burden of Disease Study 2019. J Clin Periodontol. 2021;48(9):1165–88.

14. Schenkein HA, Papapanou PN, Genco R, Sanz M. Mechanisms underlying the association between periodontitis and atherosclerotic disease. Periodontol 2000. 2020;83(1):90–106.

15. Swastini DA, Wiryanthini IAD, Ariastuti NLP, Muliantara A. Atherosclerosis Prediction with High Sensitivity C-Reactive Protein (hs-CRP) and Related Risk Factor in Patient with Dyslipidemia. Open Access Maced J Med Sci. 2019 Nov 14;7(22):3887–90.

16. Bauersachs R, Zeymer U, Brière JB, Marre C, Bowrin K, Huelsebeck M. Burden of Coronary Artery Disease and Peripheral Artery Disease: A Literature Review. Cardiovasc Ther. 2019 Nov 26;2019:1–9.

17. Rafieian-Kopaei M, Setorki M, Doudi M, Baradaran A, Nasri H. Atherosclerosis: Process, Indicators, Risk Factors and New Hopes. Int J Prev Med. 2014 Aug;5(8):927–46.

18. Schenkein HA, Loos BG. Inflammatory mechanisms linking periodontal diseases to cardiovascular diseases. J Clin Periodontol. 2013 Apr 1;40(s14):S51–69.

19. Libby P. Inflammation and cardiovascular disease mechanisms. Am J Clin Nutr. 2006;83(2):456S-460S.

20. Hayashi C, Gudino CV, Gibson III FC, Genco CA. REVIEW: Pathogen-induced inflammation at sites distant from oral infection: bacterial persistence and induction of cell-specific innate immune inflammatory pathways. Mol Oral Microbiol. 2010 Oct 1;25(5):305–16.

21. Wojtkowska A, Zapolski T, Wysokińska-Miszczuk J, Wysokiński AP. The inflammation link between periodontal disease and coronary atherosclerosis in patients with acute coronary syndromes: case–control study. BMC Oral Health. 2021 Jan 6;21(1):5.

22. Gupta M, Chaturvedi R, Jain A. Role of cardiovascular disease markers in periodontal infection: Understanding the risk: Official Publication of Indian Society for Dental Research. Indian J Dent Res. 2015;26(3):231–6.

23. Hasturk H, Kantarci A. Activation and resolution of periodontal inflammation and its systemic impact. Periodontol 2000. 2015 Oct 1;69(1):255–73.

24. Libby P, Ridker PM, Maseri A. Inflammation and Atherosclerosis. Circulation. 2002 Mar 5;105(9):1135–43.

25. Pant S, Deshmukh A, GuruMurthy GS, Pothineni NV, Watts TE, Romeo F, et al. Inflammation and Atherosclerosis—Revisited. J Cardiovasc Pharmacol Ther. 2014 Mar 1;19(2):170–8.

26. Peters SAE, Colantonio LD, Dai Y, Zhao H, Bittner V, Farkouh ME, et al. Trends in Recurrent Coronary Heart Disease After Myocardial Infarction Among US Women and Men Between 2008 and 2017. Circulation. 2021 Feb 16;143(7):650–60.

27. Australina Bureau of Statistics. Causes of Death, Australia, 2021 | Australian Bureau of Statistics [Internet]. Causes of Death, Australia. [cited 2022 Oct 27]. Available from: https://www.abs.gov.au/statistics/health/causes-death/causes-death-australia/latest-release#australia-s-leading-causes-of-death-2021

28. Cardiovascular diseases (CVDs) [Internet]. World Health Organisation - Cardiovascular diseases (CVDs). 2021 [cited 2021 Oct 25]. Available from: https://www.who.int/news-room/fact-sheets/detail/cardiovascular-diseases-(cvds)

29. Larvin H, Kang J, Aggarwal VR, Pavitt S, Wu J. Risk of incident cardiovascular disease in people with periodontal disease: A systematic review and meta-analysis. Clin Exp Dent Res. 2021 Feb 1;7(1):109–22.

30. Gasner NS, Schure RS. Periodontal Disease. In: StatPearls [Internet]. Treasure Island (FL): StatPearls Publishing; 2022 [cited 2022 Nov 3]. Available from: http://www.ncbi.nlm.nih.gov/books/NBK554590/

31. Scannapieco FA, Gershovich E. The prevention of periodontal disease—An overview. Periodontol 2000. 2020;84(1):9–13.

32. Casanova L, Hughes FJ, Preshaw PM. Diabetes and periodontal disease: a two-way relationship. Br Dent J. 2014 Oct;217(8):433–7.

33. de Oliveira C, Watt, R, Hamer M. Toothbrushing, inflammation, and risk of cardiovascular disease: results from Scottish Health Survey. BMJ. 2010;340(c2451).

34. Park SY, Kim SH, Kang SH, Yoon CH, Lee HJ, Yun PY, et al. Improved oral hygiene care attenuates the cardiovascular risk of oral health disease: a population-based study from Korea. Eur Heart J. 2019 Apr 7;40(14):1138–45.

35. Gallagher A, Sowinski J, Bowman J, Barrett K, Patel K, Bosma ML, et al. The Effect of Brushing Time and Dentifrice on Dental Plaque Removal in vivo. 2009;83(3):6.

36. Janakiram C, Taha F, Joe J. The Efficacy of Plaque Control by Various Toothbrushing Techniques-A Systematic Review and Meta-Analysis. J Clin Diagn Res [Internet]. 2018;12(11). Available from: https://jcdr.net/article_fulltext.asp?issn=0973-709x&year=2018&volume=12&issue=11&page=ZE01&issn=0973-709x&id=12204

37. Grender J, Ram Goyal C, Qaqish J, Adam R. An 8‐week randomized controlled trial comparing the effect of a novel oscillating‐rotating toothbrush versus a manual toothbrush on plaque and gingivitis. Int Dent J. 2020;70(S1):S7–15.

38. Klukowska M, Grender JM, Conde E, Goyal CR. A 12-week clinical comparison of an oscillating-rotating power brush versus a marketed sonic brush with self-adjusting technology in reducing plaque and gingivitis. J Clin Dent. 2013;24(2):55–61.

39. Yaacob M, Worthington H, Deacon S, Deery C, Walmsley A, Robinson P, et al. Powered versus manual toothbrushing for oral health. Cochrane Database Syst Rev [Internet]. 2014;(6). Available from: https://doi.org//10.1002/14651858.CD002281.pub3

40. Wilkins E, Lyle D. Interdental Care and Irrigation. In: Clinical Practice of the Dental Hygienist. 11th ed. Philadelphia: Lippincott Williams & Wilkins; 2013. p. 408–22.

41. Ng E, Lim LP. An Overview of Different Interdental Cleaning Aids and Their Effectiveness. Dent J. 2019 Jun 1;7(2):56.

42. Worthington H, MacDonald L, Poklepovic Pericic T, Sambunjak D, Johnson T, Imai P, et al. Home use of interdental cleaning devices, in addition to toothbrushing, for preventing and controlling periodontal diseases and dental caries. Cochrane Database Syst Rev [Internet]. 2019;(4). Available from: https://doi.org//10.1002/14651858.CD012018.pub2

43. Nakre PD, Harikiran AG. Effectiveness of oral health education programs: A systematic review. J Int Soc Prev Community Dent. 2013;3(2):103–15.

44. King S, Church L, Garde S, Chow CK, Akhter R, Eberhard J. Targeting the reduction of inflammatory risk associated with cardiovascular disease by treating periodontitis either alone or in combination with a systemic anti-inflammatory agent: protocol for a pilot, parallel group, randomised controlled trial. BMJ Open. 2022 Nov 1;12(11):e063148.

45. Hussain A, Al Rifai M, Khalid U, Virani SS. Cardiovascular Risk Assessment in Primary Prevention. In: Shapiro MD, editor. Cardiovascular Risk Assessment in Primary Prevention [Internet]. Cham: Springer International Publishing; 2022. p. 3–19. Available from: https://doi.org/10.1007/978-3-030-98824-1_1

46. Department of Health, State Government of Victori. Heart disease - know your risk [Internet]. Better Health Channel Victoria. 2022. Available from: https://www.betterhealth.vic.gov.au/health/conditionsandtreatments/heart-disease-risk-factors#risk-factors

47. 8 Things You Can Do to Prevent Heart Disease and Stroke | American Heart Association [Internet]. [cited 2022 Sep 22]. Available from: https://www.heart.org/en/healthy-living/healthy-lifestyle/prevent-heart-disease-and-stroke

48. Prevent Heart Disease | cdc.gov [Internet]. [cited 2022 Sep 22]. Available from: https://www.cdc.gov/heartdisease/prevention.htm

49. Coronary heart disease - Prevention - NHS [Internet]. [cited 2022 Sep 22]. Available from: https://www.nhs.uk/conditions/coronary-heart-disease/prevention/

50. Right to health [Internet]. Attorney-General’s Department. [cited 2022 Nov 1]. Available from: https://www.ag.gov.au/rights-and-protections/human-rights-and-anti-discrimination/human-rights-scrutiny/public-sector-guidance-sheets/right-health

51. Human rights and health [Internet]. [cited 2022 Nov 1]. Available from: https://www.who.int/news-room/fact-sheets/detail/human-rights-and-health

52. Oral Health Monitoring Group. Healthy Mouths Healthy Lives - Australia’s National Oral Health Plan 2015-2024 [Internet]. COAG Health Council 2015; 2015 [cited 2022 Mar 11]. Available from: https://www.health.gov.au/sites/default/files/documents/2022/04/healthy-mouths-healthy-lives-australia-s-national-oral-health-plan-2015-2024-australia-s-national-oral-health-plan-2015-2024.pdf

53. Fields LB. Oral care intervention to reduce incidence of ventilator-associated pneumonia in the neurologic intensive care unit. J Neurosci Nurs. 2008;40(5):291–8.

54. Dagnew ZA, Abraham IA, Beraki GG, Mittler S, Achila OO, Tesfamariam EH. Do nurses have barriers to quality oral care practice at a generalized hospital care in Asmara, Eritrea? A cross-sectional study. BMC Oral Health. 2020 May 20;20(1):149.

55. Bonetti D, Hampson V, Queen K, Kirk D, Clarkson J, and LY. Improving oral hygiene for patients. Nurs Stand. 2014;29(19):44–50.

56. McNally ME, Martin-Misener R, Wyatt CCL, McNeil KP, Crowell SJ, Matthews DC, et al. Action Planning for Daily Mouth Care in Long-Term Care: The Brushing Up on Mouth Care Project. Nurs Res Pract. 2012 Apr 5;2012:e368356.

57. Lupi SM, Pascadopoli M, Maiorani C, Preda C, Trapani B, Chiesa A, et al. Oral Hygiene Practice among Hospitalized Patients: An Assessment by Dental Hygiene Students. Healthcare. 2022 Jan 6;10(1):115.

58. Wallace JP, Blinkhorn FA, Blinkhorn AS. Dental hygiene students’ views on a service-learning residential aged care placement program. J Dent Hyg. 2014;88(5):309–15.

59. Girestam Croonquist C, Dalum J, Skott P, Sjögren P, Wårdh I, Morén E. Effects of Domiciliary Professional Oral Care for Care-Dependent Elderly in Nursing Homes – Oral Hygiene, Gingival Bleeding, Root Caries and Nursing Staff’s Oral Health Knowledge and Attitudes. Clin Interv Aging. 2020 Aug 6;15:1305–15.

60. Weintraub JA, Zimmerman S, Ward K, Wretman CJ, Sloane PD, Stearns SC, et al. Improving Nursing Home Residents’ Oral Hygiene: Results of a Cluster Randomized Intervention Trial. J Am Med Dir Assoc. 2018 Dec;19(12):1086–91.

61. Amerine C, Boyd L, Bowen DM, Neill K, Johnson T, Peterson T. Oral health champions in long-term care facilities-a pilot study. Spec Care Dentist. 2014;34(4):164–70.

62. Wallace J, Mohammadi J, Wallace L, Taylor J. Senior Smiles: preliminary results for a new model of oral health care utilizing the dental hygienist in residential aged care facilities. Int J Dent Hyg. 2016;14(4):284–8.

63. Wallace J. The ‘Senior Smiles’ model of preventative oral health care in Residential Aged Care Facilities. Aust N Z J Dent Oral Health Ther. 2017;6(2):1 and 4.

64. Gosney M, Martin MV, Wright AE. The role of selective decontamination of the digestive tract in acute stroke. Age Ageing. 2006 Jan 1;35(1):42–7.

65. Charteris P, Kinsella T. The Oral Care Link Nurse: a facilitator and educator for maintaining oral health for patients at the Royal Hospital for Neuro-disability. Spec Care Dentist. 2001 Mar 1;21(2):68–71.

66. de Mey L, Çömlekçi C, de Reuver F, van Waard I, van Gool R, Scheerman JFM, et al. Oral Hygiene in Patients With Severe Mental Illness: A Pilot Study on the Collaboration Between Oral Hygienists and Mental Health Nurses. Perspect Psychiatr Care. 2016 Jul 1;52(3):194–200.

67. Omori C, Ekuni D, Ohbayashi Y, Miyake M, Morita M. Quasi-Randomized Trial of Effects of Perioperative Oral Hygiene Instruction on Inpatients with Heart Diseases Using a Behavioral Six-Step Method. Int J Environ Res Public Health. 2019 Nov 1;16(21):4252.

68. Lam OL PhD, McMillan AS PhD, Samaranayake LP FRCPath, Li LS MBBS, McGrath C PhD. Randomized Clinical Trial of Oral Health Promotion Interventions Among Patients Following Stroke. Arch Phys Med Rehabil. 2013;94(3):435–43.

69. Cinar AB, Schou L. Impact of empowerment on toothbrushing and diabetes management. Oral Health Prev Dent. 2014;12(4):337–44.

70. Almomani F, Williams K, Catley D, Brown C. Effects of an Oral Health Promotion Program in People with Mental Illness. J Dent Res. 2009;88(7):648–52.

71. Kuo MW, Yeh SH, Chang HM, Teng PR. Effectiveness of oral health promotion program for persons with severe mental illness: a cluster randomized controlled study. BMC Oral Health. 2020;20(1):290–290.

72. Silverstein LS, Haggerty C, Sams L, Phillips C, Roberts MW. Impact of an oral health education intervention among a group of patients with eating disorders (anorexia nervosa and bulimia nervosa). J Eat Disord. 2019;7(1):29–29.

73. Yoshii H, Kitamura N, Akazawa K, Saito H. Effects of an educational intervention on oral hygiene and self-care among people with mental illness in Japan: a longitudinal study. BMC Oral Health. 2017;17(1):81–81.

74. Sanchez P, Everett B, Salamonson Y, Redfern J, Ajwani S, Bhole S, et al. The oral health status, behaviours and knowledge of patients with cardiovascular disease in Sydney Australia: a cross-sectional survey. BMC Oral Health. 2019 Jan 11;19(1):12.

75. Sanchez P, Everett B, Salamonson Y, Ajwani S, Bhole S, Bishop J, et al. Oral health and cardiovascular care: Perceptions of people with cardiovascular disease. PLOS ONE. 2017 Jul 20;12(7):e0181189.

76. Oudkerk Pool MD, Hooglugt JLQ, Schijven MP, Mulder BJM, Bouma BJ, de Winter RJ, et al. Review of Digitalized Patient Education in Cardiology: A Future Ahead? Cardiology. 2021;146(2):263–71.

77. McIntyre D, Thiagalingam A, Klimis H, Huben AV, Marschner S, Chow CK. Education on cardiac risk and CPR in cardiology clinic waiting rooms: a randomised clinical trial. Heart. 2021 Oct 1;107(20):1637–43.

78. Tait AR, Voepel-Lewis T, Chetcuti SJ, Brennan-Martinez C, Levine R. Enhancing Patient Understanding of Medical Procedures: Evaluation of an Interactive Multimedia Program with In-line Exercises. Int J Med Inf. 2014 May;83(5):376–84.

79. Rossi MJ, Guttmann D, MacLennan MJ, Lubowitz JH. Video informed consent improves knee arthroscopy patient comprehension. Arthrosc J Arthrosc Relat Surg Off Publ Arthrosc Assoc N Am Int Arthrosc Assoc. 2005 Jun;21(6):739–43.

80. McNab M, Skapetis T. Why video health education messages should be considered for all dental waiting rooms. PLOS ONE. 2019 Jul 16;14(7):e0219506.

81. Lange D. Accessory treatment in systemic periodontitis management. Zahnärztliche Welt. 1975;8:366–74.

82. Find a cardiac rehabilitation service near you | The Heart Foundation [Internet]. [cited 2022 Oct 25]. Available from: https://www.heartfoundation.org.au/bundles/support/cardiac-services-directory

83. Mühlemann H, Son S. Gingival sulcus bleeding--a leading symptom in initial gingivitis. Helv Odontol Acta. 1971;15(2):107–13.

84. Harris PA, Taylor R, Minor BL, Elliott V, Fernandez M, O’Neal L, et al. The REDCap consortium: Building an international community of software platform partners. J Biomed Inform. 2019 Jul;95:103208.

85. Löe H, Theilade E, Jensen SB. Experimental Gingivitis in Man. J Periodontol. 1965;36(3):177–87.

86. Wyche C. Indices and Scoring Methods. In: Wilkins E, editor. Clinical Practice of the Dental Hygienist. 11th ed. Philadelphia: Lippincott Williams & Wilkins; 2013. p. 311–35.

87. Ziebolz D, Herz A, Brunner E, Hornecker E, Mausberg RF. Individual versus group oral hygiene instruction for adults. Oral Health Prev Dent. 2009;7(1):93–9.

88. iPerioPal. SBI/API Index Sulcus Bleeding Index (SBI and Approximal Plaque Index (API) [Internet]. Available from: https://www.iperiopal.com/sbi-api-index/

89. Willmann D, Neild-Gehrig J. Clinical Periodontal Assessment. In: Foundations of Periodontics for the Dental Hygienist. Third. Philadelphia: Wolters Kluwer | Lippincott WIlliams & Wilkins; 2011. p. 321–41.

90. Adams R. Qualified nurses lack adequate knowledge related to oral health, resulting in inadequate oral care of patients on medical wards. J Adv Nurs. Accepted for publication 6 October 1995. 1996;24(3):552–60.

91. Cianetti S, Anderini P, Pagano S, Eusebi P, Orso M, Salvato R, et al. Oral Health Knowledge Level of Nursing Staff Working in Semi-Intensive Heart Failure Units. J Multidiscip Healthc. 2020 Feb 12;13:165–73.

92. Gibney J, Wright C, Sharma A, Naganathan V. Nurses’ knowledge, attitudes, and current practice of daily oral hygiene care to patients on acute aged care wards in two Australian hospitals. Spec Care Dentist. 2015 Nov 1;35(6):285–93.

93. Sanchez P, Everett B, Salamonson Y, Ajwani S, Bhole S, Bishop J, et al. Perceptions of cardiac care providers towards oral health promotion in Australia. Coll R Coll Nurs Aust. 2018;25(5):471–8.
